# Mechanical strain determines the site-specific localization of inflammation and tissue damage in arthritis

**DOI:** 10.1038/s41467-018-06933-4

**Published:** 2018-11-05

**Authors:** Isabelle Cambré, Djoere Gaublomme, Arne Burssens, Peggy Jacques, Nadia Schryvers, Amélie De Muynck, Leander Meuris, Stijn Lambrecht, Shea Carter, Pieter de Bleser, Yvan Saeys, Luc Van Hoorebeke, George Kollias, Matthias Mack, Paul Simoens, Rik Lories, Nico Callewaert, Georg Schett, Dirk Elewaut

**Affiliations:** 1 Unit for Molecular Immunology and Inflammation, VIB Inflammation Research Center (IRC), Technologiepark 927, 9052 Ghent, Belgium; 2 Department of Rheumatology, Ghent University, Ghent University Hospital, De Pintelaan 185, 9000 Ghent, Belgium; 3 Department of Orthopaedics and Traumatology, Ghent University Hospital, De Pintelaan 185, 9000 Ghent, Belgium; 4 UGCT-Department of Physics and Astronomy, Ghent University, Proeftuinstraat 86, 9000 Ghent, Belgium; 5 Unit of Medical Biotechnology, VIB Inflammation Research Center (IRC), VIB, Technologiepark 927, 9052 Ghent, Belgium; 6 Laboratory for Protein Biochemistry and Biomolecular Engineering, Department of Biochemistry and Microbiology, Ghent University, Technologiepark 927, 9000 Ghent, Belgium; 7 Department of Development and Regeneration, Laboratory of Tissue Homeostasis and Disease, Skeletal Biology and Engineering Research Center, KU Leuven, Herestraat 49, 3000 Leuven, Belgium; 8 Division of Rheumatology, University Hospitals Leuven, Herestraat 49, 3000 Leuven, Belgium; 9 Data Mining and Modeling for Biomedicine, VIB Center for Inflammation Research, Technologiepark 927, 9052 Ghent, Belgium; 10 Division of Immunology, Biomedical Sciences Research Center ‘Alexander Fleming’, 34, Fleming Street, 16672 Vari, Attica Greece; 11 Department of Experimental Physiology, School of Medicine, National and Kapodistrian University of Athens, 75 Mikras Asias Street, 11527 Goudi, Athens Greece; 12 Deparment of Internal Medicine II – Nephrology, University Hospital Regensburg, 93402 Regensburg, Germany; 13 Department of Morphology, Faculty of Veterinary Medicine, Ghent University, Salisburylaan 133, 9820 Merelbeke, Belgium; 14 Department of Internal Medicine 3, Rheumatology and Immunology, Friedrich Alexander University of Erlangen-Nuremberg and Universitatsklinikum, Ulmenweg 18, 91054 Erlangen, Germany

## Abstract

Many pro-inflammatory pathways leading to arthritis have global effects on the immune system rather than only acting locally in joints. The reason behind the regional and patchy distribution of arthritis represents a longstanding paradox. Here we show that biomechanical loading acts as a decisive factor in the transition from systemic autoimmunity to joint inflammation. Distribution of inflammation and erosive disease is confined to mechano-sensitive regions with a unique microanatomy. Curiously, this pathway relies on stromal cells but not adaptive immunity. Mechano-stimulation of mesenchymal cells induces CXCL1 and CCL2 for the recruitment of classical monocytes, which can differentiate into bone-resorbing osteoclasts. Genetic ablation of *CCL2* or pharmacologic targeting of its receptor CCR2 abates mechanically-induced exacerbation of arthritis, indicating that stress-induced chemokine release by mesenchymal cells and chemo-attraction of monocytes determines preferential homing of arthritis to certain hot spots. Thus, mechanical strain controls the site-specific localisation of inflammation and tissue damage in arthritis.

## Abstract (web summary)

Pro-inflammatory factors implicated for the onset of arthritis often have systematic effects, yet arthritis symptoms are mostly limited to the joints. Here the authors show that mechanical strain at the joints promotes the recruitment of monocyte and their differentiation into bone-eroding osteoclast to contribute this tissue specificity.

## Introduction

While many of the molecular inflammatory processes leading to arthritis have been unravelled in the last two decades, the reason why inflammation homes to the joints, affects highly distinct anatomical regions and conveys a patchy clinical pattern affecting only some joints is still enigmatic. Key pro-inflammatory molecular pathways identified to induce arthritis such as TNFα, IL-1, IL-6, and IL-17/23 rather act systemically in triggering the onset and progression of the disease but give little hint why arthritis affects certain joints more often than others^1^. Also, master switches of immune regulation such as TGF-beta and IL-10 and those triggering resolution of arthritis like IL-9 do not provide a conclusive answer why the joints and not other organs are primarily targeted by the inflammatory process and why certain anatomical regions are more often affected than others^2^. Nonetheless, advanced imaging studies in humans have unequivocally shown that inflammation of joints, neighboring and around the joints affects certain predilection sites in the joints leading to a patchy pattern of the disease^3,4^.

Therefore, new concepts are required explaining localisation of inflammation and bone damage in diseases such as arthritis. The answers to these questions, however, may not exclusively lie in pathways related to the immune response. Notably the joints resemble unique sites for the integration of mechanical forces and biological signals. This feature allows the joints and adjacent tissue such as the bones to functionally adapt to changes in physical activity^5–7^. Hence, biomechanical forces may provide an elegant explanation for organ-specific and site-specific inflammation and bone damage as it is typically observed in experimental and human arthritis^8^. However, up till now, data supporting the concept that biomechanical forces determine the site-specificity of inflammation and bone disease in arthritis are scarce as no systematic investigations of the micro-anatomical and functional factors have been conducted.

Here we reveal that biomechanical loading acts as a decisive factor in determining the transition from systemic autoimmunity to localized joint inflammation. Hind paw unloading prevents the onset of collagen-induced arthritis (CIA) without impairing the induction of anti-collagen specific antibodies. Conversely, excess mechanical load by voluntary running accelerates the onset of arthritis induced by passive transfer of anti-collagen specific antibodies. Importantly, this mechano-sensitive pathway for induction of arthritis does not rely on adaptive immunity. By contrast, mechano-stimulation of mesenchymal cells induces the production of CXCL1 and CCL2 and recruitment of classical monocytes that may differentiate into bone-resorbing osteoclasts. Accordingly, the first bone erosions develop at mechano-sensitive zones both in mice and men. In sum, our data implicate a crucial role for mechanostress as a driver for the conversion of the autoimmune to tissue localisation of arthritis and explain the particular anatomical pattern of bone disease in arthritis.

## Results

### Mechanostress controls arthritis onset and effector phase

**Fig. 1:**
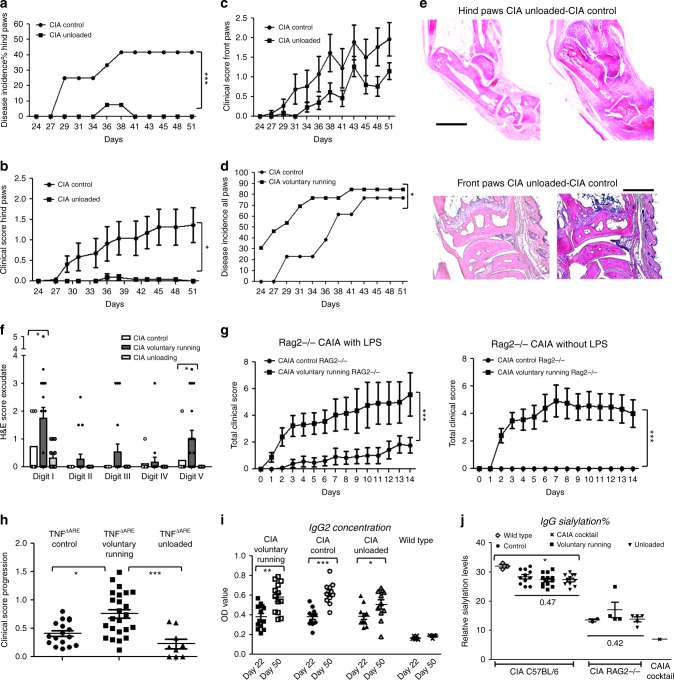
Mechanical loading controls onset of joint inflammation in active (CIA) and passive (CAIA) induced arthrits. **a**, **b** Respectively incidence and clinical scores of collagen-induced arthritis (CIA) in C57BL/6 mice at hind paws in unloaded vs. control conditions; survival analysis, mixed models repeated measurements (MMRM); *n* = 13/groups. **c** Front paws clinical scores in CIA unloaded mice vs. controls are similar, MMRM, *n* = 13/group. **d** Disease incidence (all paws included) of controls compared to voluntary running mice, survival analysis, *n* = 13/group. **e** H&E staining of the hind paws from unloaded vs. loaded CIA mice (CIA control) shows respectively absence and presence of inflammation. The front paws show comparable inflammation at the wrist joint of both groups. Scale bar hind paws 1 mm, front paws 0.25 mm. **f** H&E staining of hind paws shows more inflammation of voluntary running mice at digit I and digit V compared to control and unloaded mice, Mann–Whitney-*U* (MWU). **g** Collagen antibody induced arthritis (CAIA) treated Rag2−/− mice with or without lipopolysaccharide (LPS). Total clinical score of CAIA of voluntary running vs. control conditions is shown, MMRM, *n* = 6–13 mice/group, pooled data of three independent experiments for with LPS condition. **h** Disease progression of TNF^∆ARE^ mice during 2–3 weeks in condition (control (*n* = 16); voluntary running (*n* = 24); unloaded (*n* = 8)). Increased progression by enhanced activity (voluntary running) and slower progression by diminished activity, pooled data of three independent experiments, Kruskall-Wallis (KW) and post-test. **i** Anti- Collagen type 2 IgG2a levels at day 22 and 51 after the CIA initiation shows significant increase of these antibodies throughout the experiment regardless of the loading conditions, MWU, *n* = 13/group. **j** IgG relative sialylation levels, is shown for C57BL/6 wild type (*n* = 3; day 35) mice, CIA C57BL/6 (*n* = 11/condition, day 35) and CAIA RAG2−/− (*n* = 2–5/condition, day14) mice under different mechanical loading conditions which induced no differences in IgG sialylation level. In contrast, CIA caused a decreased sialylation level compared to wild type C57BL/6 samples, KW (day 35). The sialylation level of the CAIA cocktail is shown to be very low. Error bars show the mean ± s.e.m., if not mentioned otherwise data are representative for two independent experiments.*p < 0.05, **p < 0.01, ***p < 0.001

In an effort to examine the impact of biomechanical factors in arthritis we performed hind limb unloading experiments vs. voluntary running and respective control conditions in CIA a standard model of experimental inflammatory arthritis. Joint disease was induced by immunizing C57BL/6 mice with heterologous collagen type II. Animals were exposed afterwards to different loading conditions. At day 22 after the primary immunization, mice were either tail-suspended to avoid hind limb loading (CIA unloaded) or kept in control cages (CIA control) for a period of 4 weeks. None of the unloaded mice developed clinical arthritis at their unloaded hind paws. By contrast, arthritis incidence of hind paws of control mice steadily increased throughout the experiment (Fig. 1a). Likewise hind paw arthritis scores were significantly different between CIA unloaded mice and controls (Fig. 1b). Histological assessment of hind paws showed significant differences in total inflammation scores between unloaded and control mice, most strikingly around the Achilles tendon and the ankle joint (Fig. 1e). As an internal control, we also evaluated the incidence and frequency of arthritis at the loaded front paws. In marked contrast, the front paws exhibited a similar degree of arthritis in CIA unloaded mice and controls (Fig. 1d). In line with this observation, synovial joint inflammation could be readily observed in both treatments in the front paws of both treatments (Fig. 1e lower panel) but not in the hind paws (Fig. 1e upper panel).

In order to evaluate the impact of increased biomechanical loading, experiments were designed to promote voluntary running by housing animals in cages with running wheels. To control for putative differences in distances, we monitored the average distances by registering the number of running wheel spins per day per mouse. In non-arthritic control mice average running distance was 3.5 km per day (mean of 8 C57BL/6 mice). Mice were induced for CIA and allowed to run voluntarily (CIA voluntary running) from day 22 after CIA initiation until they were sacrificed at day 51, while controls were kept in cages without running wheels (CIA control). As anticipated, CIA mice had a significantly lower mean running distance/day compared to non-arthritic control mice (mean 2.5 km/day). A marked accelerated onset was noted in voluntary running group vs. the control group (Fig. 1e). Enhanced arthritis was mirrored by significantly more inflammation in the forefoot of the hindpaw, mainly at digit I and digit V (Fig. 1f). Voluntary running conditions were also initiated at earlier time points starting 1 day after primary immunization which showed comparable results (Supplementary Figure1a).

To test whether biomechanical force-induced homing of inflammation to the joints represents a common principle of arthritis we extended our experiments to another model of arthritis, collagen antibody induced arthritis (CAIA), which only mimics the effector phase of the disease. CAIA was induced in T-cell and B-cell deficient Rag2−/− mice challenged by lipopolysaccharide (LPS) and anti-collagen type 2 antibody (CAIA) cocktail. Voluntary running markedly aggravated CAIA in Rag2−/− mice suggesting that mechanical strain primarily impacts the effector phase of arthritis independently of adaptive immunity (Fig. 1g left panel). In the absence of prior LPS stimulation typically required to induce CAIA (Fig. 1g right panel) only voluntarily running mice developed arthritis suggesting that the effector phase of arthritis does not require exogenous TLR ligand engagement under running conditions. Finally, the aggravating role of voluntary running was also confirmed in a third model of arthritis. Hence, in an TNF-overexpression model of arthritis (TNF^***Δ****ARE*^ mice), which resembles an effector model of arthritis with features partly resembling human spondyloarthritis (SpA), mice that had been voluntarily running developed significantly exacerbated disease (Fig. 1h)). Thus, mechanical loading controls the threshold for onset and severity of arthritis in a variety of different experimental arthritis models including those mimicking the effector phase of disease.

### Mechanostress does not affect autoantibody production

One of the prominent features of some forms of experimental arthritis and human rheumatoid arthritis (RA) is the development of humoral autoimmunity before disease onset. This process is reflected by the development of anti-collagen type 2 antibodies before the onset of CIA. We therefore sought to evaluate whether biomechanical loading impacts the levels of anti-collagen antibodies during CIA. Therefore, anti-collagen type 2 IgG1 and IgG2 concentrations were determined at day 22 and at the end of the experiment. Anti-collagen antibodies increased significantly during the course of CIA regardless of biomechanical loading regime (Fig. 1i; Supplementary Figure 1b). Recently, it was shown that IL-23 unlocks autoantibody activity through modulating IgG glycosylation patterns by decreasing sialylation of arthritogenic antibodies^9^. This parallels changing galactosylation during autoimmune arthritis^10^. In theory, biomechanical loading could affect galactosylation as well as sialylation. However, as shown in Fig. 1j, no differences in relative sialylation levels in response to mechanical loading were found in CIA and CAIA. Similar findings were observed in wild-type mice (voluntary running compared to control, Supplementary Figure 1c). Additionally, increased or decreased biomechanical loading did not affect galactosylation of IgG glycans in either CIA, CAIA or wild-type mice (Supplementary Figure 1d). Thus, while biomechanical forces have a dramatic impact on the effector phase of arthritis, they do not interfere with systemic signs of autoimmunity nor with the levels of sialylation or galactosylation of Fc N-glycans on arthritogenic antibodies.

### Loading promotes erosions at biomechanically-exposed areas

**Fig. 2:**
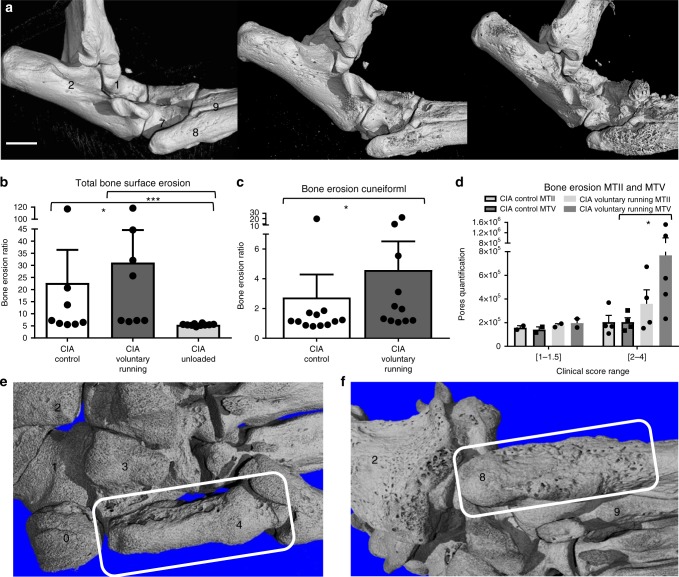
Site-specific inflammation induced erosions appear in response to mechanical stress. **a** Lateral view of micro-CT scans of CIA under voluntary running (right panel), unloaded (left panel) or control conditions (middle panel). Displayed mice had a similar clinical arthritis score. Talus (1), calcaneus (2), cuboid bone (7), MTV (8), MTIV (9). Scale bar: 1 mm. **b** Total bone surface erosion, cumulative quantification of bones in the midfoot and hindfoot shows significant increase of erosion by biomechanical loading (cuneiform I (4), navicular bone (3), cuboid bone (7), calcaneus (2), sesamoid tarsal bone (0)). CIA in voluntary running, unloaded or control conditions *n* = 8–10, KW and post-test. **c** Surface bone erosion quantification in CIA of cuneiform I for voluntary running mice shows increase vs. controls, MWU, *n* = 8–10/group. **d** Bone erosion quantification in CIA of metatarsal V and metatarsal II under voluntary running or control conditions for mice with varying degrees of clinical arthritis scores at the scanned hind paw (range 1–4, *n* = 6–7). Voluntary running causes increased bone erosion at MTV but not at MTII, significant for range 2–4 MWU. **e** Plantar view of micro-CT of cuneiform I in CIA from voluntary running mice (white rectangle). **f** Micro-CT of MTV in CIA under voluntary running conditions (white rectangle), plantar side. Error bars show the mean ± s.e.m., data are pooled from two independent experiments *p < 0.05, ***p < 0.001

**Fig. 3:**
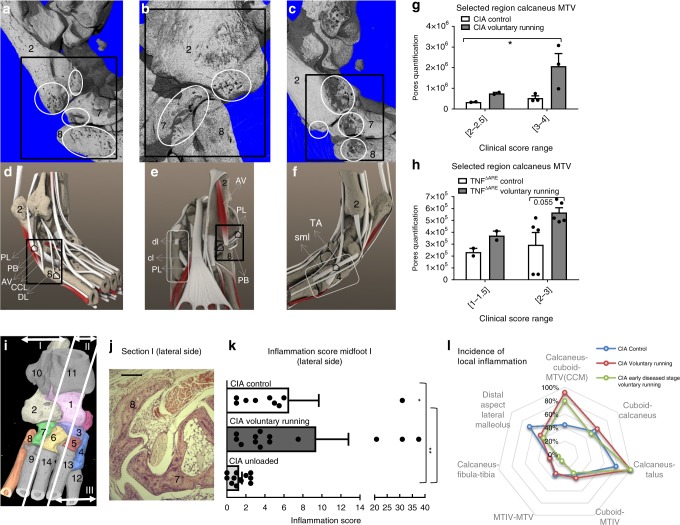
The calcaneus-cuboid-MTV (CCM) complex and cuneiform I are mechanosensitive sites. **a**, **b** Dorsolateral and plantar micro-CT views of hind paws of CIA in voluntary running mice. Black rectangle indicates CCM region, white ovals display sites of erosions. **c** Lateral micro-CT views of hind paws from TNF^∆ARE^ voluntary running mice; eroded regions are encircled. **d**–**f** Microanatomy of dorsolateral, plantar and medial side of hind paws representing all tendons, ligaments, and muscles. CCM: black rectangle, Cuneiform I: grey rectangle. Indicated tendons in the CCM region are PL peroneus longus, PB peroneus brevis, AV abductor digiti V, CCL calcaneocuboid ligament, DL extensor digitorum lateralis, TA tibialis posterior, dl distal ligament, cl cuneosesamoid ligament, sml sesamoidalMT ligament. Functional enthesis ⌂, (e.g., sites where tendon wraps around a bone pulley and associated with a tendon sheath lined by a synovial membrane, rather than insertion in the immediate vicinity of these regions). **g**, **h** Bone erosion scores in CCM region in respectively CIA and TNF^∆ARE^ mice under voluntary running or control conditions, *n* = 5; *n* = 7 each condition pooled data of two independent experiments, MWU, significance for CIA score range [2–4] and TNF^∆ARE^ score range [2–3]. **i** Schematic view of hind paws divided into three regions: I (lateral), II (axial) and III (medial). H&E sections of all regions were generated and scored accordingly. 1-Talus (light pink) 2-Calcaneus (light grey) 3-Navicular bone (purple) 4-CuneiformI (blue) 5-Cuneiform II (blown) 6-Cuneiform III (yellow) 7-Cuboid bone (green) 8-Metatarsal V (orange) 9-Metatarsal IV 10- Fibula 11-Tibia and 12-13-14-Metatarsal I-II-III. **j** H&E section in voluntary running mice: early signs of inflammation in the CCM region. Scale bar: 0.2 mm. **k** H&E micro-inflammation scores at the lateral side (region I) of the mid foot increases upon biomechanical stress, KW and post-test, significance for CIA control vs. CIA unloaded and CIA voluntary running vs. CIA unloaded. **l** Incidence of local inflammation scores at the lateral side of the midfoot and hindfoot for three groups: CIA control (blue), CIA voluntary running (red) and CIA early diseased stage voluntary running. In all conditions inflammation occurs primary at the calcaneus/talus. Voluntary running increases inflammation at several sites, most pronounced at the CCM complex. Error bars show the mean ± s.e.m., if not mentioned otherwise data are representative for two independent experiments, *p < 0.05,**p < 0.01

A common hallmark of the different forms of arthritis in animals and humans is the early induction of bone erosions, which occur at distinct articular sites. The reason for this patchy pattern of bone damage in arthritis is not well understood but biomechanical factors have always been considered to be potentially important. We therefore applied high-resolution micro-CT techniques combined with microanatomy studies to examine where bone surface erosions primarily occur in early phases of arthritis and whether biomechanical forces modulate this pattern. We initially generated 3D micro-CT scans of bones from CIA-induced mice and quantified the degree of bone erosions in the entire foot and in each individual bone of the foot. A strong impact of biomechanical loading was noted on the development of erosions. Increased loading resulted in a tendency towards more bone erosions (Fig. 2a, b), while unloading prohibited development of erosions in the midfoot and hindfoot. Surprisingly, marked topographic differences were noted in bone erosions when individual bones were examined. Voluntary loading augmented erosion in the cuneiform I bone (the most medial bone of the midfoot) while in control conditions erosions were rarely apparent at this specific site (*p* < 0.05, MWU) (Fig. 2c, e). Similar results were obtained for the 5th metatarsal bone (MTV). In striking contrast however, other sites such as 2nd metatarsal bone, were largely unaffected by the impact of altered mechanical forces. To rule out that these findings were only secondary to differences in clinical scores we matched erosion scores of individual bones with global clinical scores. As shown in Fig. 2d, e, the regional differences between MTII and V became even more pronounced with an increased degree of inflammation, suggesting that the microanatomy could account for these observations. Overall the lateral region of the midfoot formed by the calcaneus-cuboid-MTV (CCM) joints was most prone to erosions elicited by voluntary running (Fig. 3a, b, g). 3D reconstructions of CIA voluntary running and wild type (cuboid bone) are depicted in Supplementary Movie 1 and 2. A similar effect of biomechanical forces on erosion development was also observed in the TNF^∆ARE^ model (Fig. 3c, h).

Because our data highlighted that erosions develop at distinct sites exposed to biomechanical stress, we next performed a microanatomy study to investigate the basis for this link (detailed information in interactive application, Supplementary Software 1). The aforementioned CCM region is composed of two joints in close proximity of each other, allowing high-level mobility and therefore high-level biomechanical stress. The same applies to the cuneiform I-MT1 region. Curiously, a synovial pouch at the CCM region and at the Cuneiform I-MTI junction was already described in the late 90′s^11^ and we confirmed these findings by in-depth histological analysis. Tendons and ligaments attach to the bone near the joint, forming an entheseal structure that overlays the synovial membrane and forms a synovio-entheseal complex^12^. Detailed analysis of these regions showed that the CCM region contains four direct tendon attachment sites and three additional contact zones with tendons (detailed anatomical description Supplementary Note 1) (Fig. 3d–f black square). At cuneiform I we mapped 4 direct tendon attachment sites and one additional contact zone with tendons (Fig. 3d–f white rectangle). In striking contrast, the 2nd metatarsal bone contains only one single small attachment site. Taken together, these data show that mechanical forces affect bones at specific sites, these sites are characterized by high-level mobility and rich in attachment sites or contact zones of tendons.

An obvious explanation for this regional distribution of erosive disease is that inflammation shows site-specific differences. To evaluate this concept we performed in-depth histologic evaluation of joints in CIA under different loading regimens (as described in Materials and methods) focussing on the midfoot and hindfoot (Fig. 3i, j). We observed that the lateral side of the hind paw is the most commonly and most severely inflamed region. Voluntary running experiments induced a parallel increase in the mean total inflammation score at this particular region (Fig. 3k, l). We next assessed the incidence of local inflammation at lateral side of midfoot and hindfoot in CIA under control and voluntary running conditions. Two regions were most prone to increased signs of inflammation after voluntary running conditions with the CCM region being the most responsive (Fig. 3l). The data were comparable in the early vs. late stage disease and reflect the distinct pattern of bone erosion observed in the micro-CT analysis (Fig. 2e, f). Overall these data support the concept that biomechanical forces promote local inflammation at mechano-sensitive sites, which subsequently leads to bone erosions.

### Mechanostressed regions prone to erosion in human arthritis

**Fig. 4:**
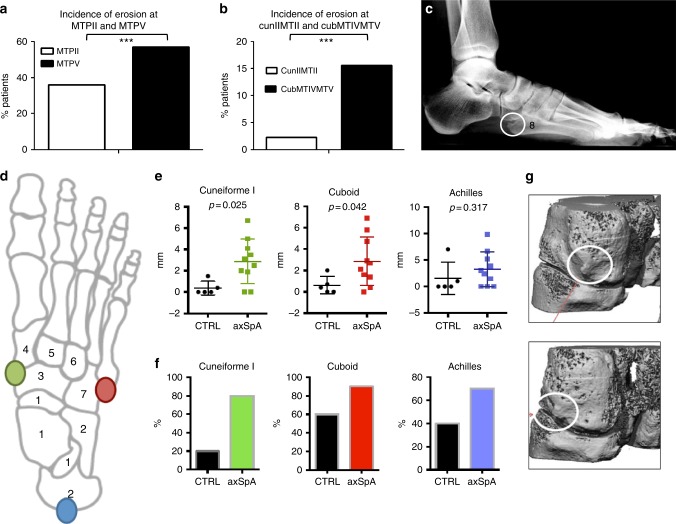
Mechano-sensitive region are prone to bone damage in human arthritis. **a**, **b** Incidence of erosion in forefoot of RA patients (*n* = 64). X-rays were scored according to the modified Sharp van de Heijde score. Comparison in erosion between the enthesis-poor MTPII and enthesis-rich MTPV joint as well as between enthesis-poor cunII-MTII and enthesis-rich cub-MTIV-MTV joint; MWU. **c** X-ray RA with erosion at base of MTV (white circle). **d** Mapping and quantification of enthesophytes was determined in patients with axial spondyloarthritis (*n* = 10) vs. healthy controls (*n* = 5). High resolution quantitative computed tomography (HR-pQCT) was conducted of feet in patients with SpA to detect hot spots of bone damage. Colored circles indicate the studied locations; green cuneiformI-navicular joint, red cuboid-MTV joint, blue calcaneus tuberosity. **f** Differences in lesion prevalence between healthy persons (black) and axSpA patients can be observed at cuneiform I (green), cuboid (red) and the Achilles tendon (blue). **e** Scores represent size of enthesophytes. axSpA patients have significant larger enthesophytes at cuneiformI (green) and cuboid bone (red), MWU. Data are shown in mm. **g** Micro-CT images of enthesis-rich cuneiform I-navicular joint (green region on figure **d**), region of enthesophyte is encircled at the medioplantar side of cuneiform I, which is exactly the attachment point of the tibialis anterior tendon. Error bars show the mean ± s.e.m., ***p < 0.001

To translate our findings to human arthritis we investigated conventional radiographs of the feet from patients with RA according to the modified Sharp-van der Heijde score^13^. These analyses showed an increased prevalence of erosions at the lateral MTV compared to the central 2nd metatarsal bone in the forefoot (Fig. 4a) and the lateral CCM region compared to the central Cuneiform II-MT base II region in the midfoot (Fig. 4b, c). These observations on the distribution of bone damage in human RA mimic the situation in mice astonishingly well and suggest a common principle in site-specific inflammation and bone damage in arthritis, which is based on biomechanical exposure of the respective region.

To extend this concept we additionally investigated the feet of human patients with another form of inflammatory arthritis for signs of site-specific bone damage. In patients with SpA inflammation is accompanied with a tissue remodelling process leading to new bone formation in the spine or the peripheral joints, which can also cause significant functional impairment. We therefore performed a high-resolution peripheral quantitative computed tomography (HR-pQCT) investigation of the feet of patients with SpA to detect hot-spots of bone damage. We could again confirm the aforementioned pattern of bone lesions with preferential affection of the CCM region as well as the cuneiform I-MT1 region. In addition, the insertion of the Achilles tendon on the heel bone was also affected in patients with SpA (Fig. 4d). There was a significant difference in the frequency of lesions in SpA patients vs. controls at all these sites (Fig. 4f). Additionally, significantly larger entheseophytes occurred in the CCM and cuneiform I -MT1 regions of SpA patients with a similar tendency at the Achilles tendon insertion (Fig. 4e, g). These findings highlight a common principle of biomechanical forces in determining the site-specific origin of arthritis in mice and humans.

### Mechanostressed stroma recruits classical monocytes locally

**Fig. 5:**
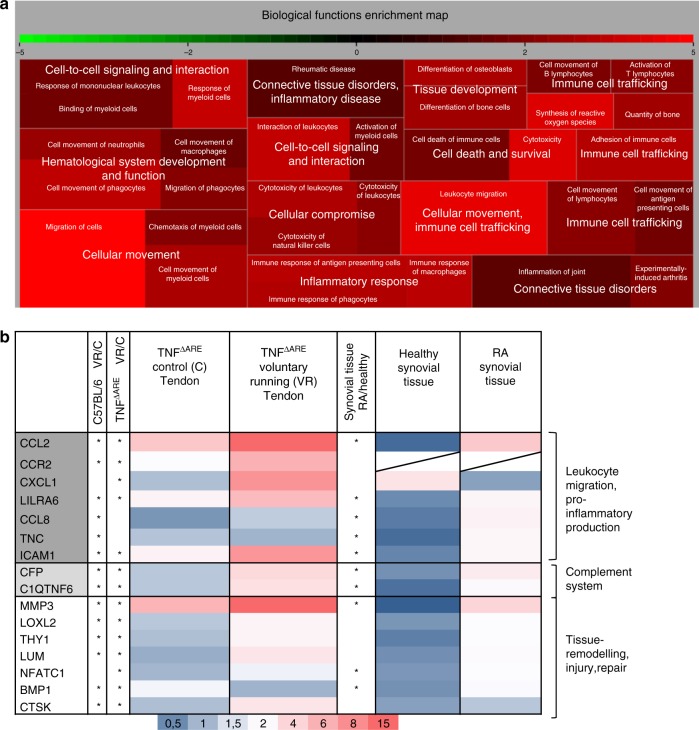
Mechanostretch induced expression of proinflammatory cytokines, chemokines, and tissue remodelling in Achilles tendon. **a** Biological deregulated functions of achilles tendon samples from C57BL/6 voluntary running compared to control mice are shown in a treemap. The most important biological deregulated functions include cellular movement, cell to cell signalling and interaction and connective tissue (and bone) disorders and development. Colors indicate the activation *z*-score of processes: activated processes are colored with a differend kinds of red according to degrees of activation (from low (dark red) to high level (light red)), while inhibited processes are colored in green. Sizes of squares are proportional to −log (*p*-values). **b** Expression validation of genes that are highly upregulated in voluntary running deregulated biological functions (C57BL/6 tendons) in TNF^∆ARE^ micro-dissected tendons under voluntary running or control conditions and validation in healthy- and RA synovial tissue. Increase in pro-inflammatory cytokine and chemokine induction related to myeloid cell recruitment as well as genes related to osteoclasts, tissue remodeling or complement regulatory pathway under running conditions. Color scale is defined below figure ranging from dark blue (low expression values) to magneta (high expression values), MWU, * shows significance meaning *p* < 0.05 or more

To unravel the underlying mechanism of site-specific origin of arthritis in mechano-sensitive regions, we studied the crosstalk between mechanical forces, tissue resident stromal cells and haematopoietic cells, notably myeloid cells. To this end we micro-dissected Achilles tendons of healthy mice (C57BL/6) exposed to voluntary running or control regimens for 14 days to determine gene expression profiles. The microarray data of the voluntary running conditions showed upregulation of biological functions like cellular movement, cell to cell signalling and interaction and connective tissue (and bone) disorders and development (Fig. 5a). Genes that were significantly upregulated by voluntary running in these important biological deregulated functions were validated in arthritic (TNF^∆ARE^ mice) as well as in RA-patient synovial tissue samples. We found a clear increase of the chemokines *CCR2*, *CCL2*, *CXCL1* as well as pro-inflammatory mediators such as *MMP3*, *LILRA6*, *ICAM1* and osteoclast specific genes such as *CTSK*, *NFATC1,* and *LILRA6* (PIR-A) in voluntary running conditions in both C57BL/6 and TNF^∆ARE^ samples highlighting a more pro-inflammatory environment, increased chemotaxis and proliferation of myeloid cells and osteoclasts (Fig. 5b). A similar increase could be observed when RA synovial tissue was compared to samples from healthy subjects with the exception of *CXCL1*. Furthermore, expression of regulators of the complement system was promoted by mechanical stresses such as *CFP*, the only known positive regulator of the complement system, and *C1QTNF6* which is an endogenous complement regulator, both in healthy C57BL/6 and TNF^∆ARE^ mice. Similar results were observed in RA synovial tissue vs. healthy controls. Genes linked to tissue remodelling and repair were also enhanced in both healthy C57BL/6 and TNF^∆ARE^ mice, specifically genes linked to osteoclast-remodelling and cartilage remodelling (*CTSK*, *NFATC1* and *BMP1*). They were also upregulated in RA synovial tissue.

**Fig. 6:**
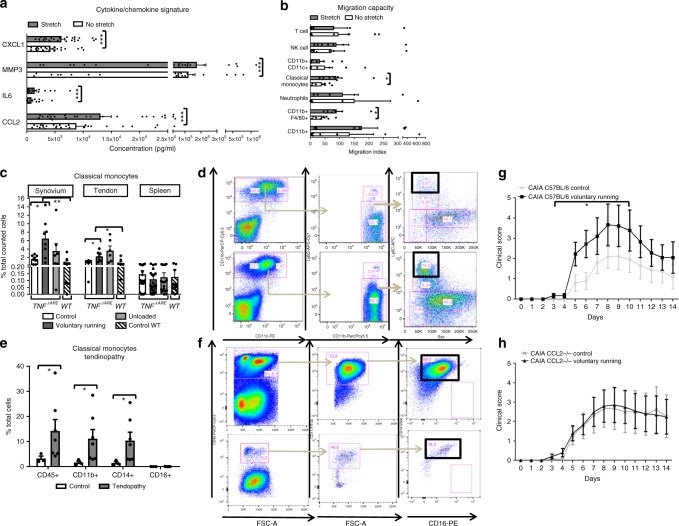
Mechanical strain in stromal cells induces recruitment of classical monocytes into mechano-sensitive regions. **a** Increased secretion of CXCL1, MMP3, IL6, and CCL2 in stromal cells (synovial- or Achilles tendon fibroblasts) upon in vitro stretch; *n* = 25 samples of individual mice, Wilcoxon. **b** Increased migration of classical monocytes and CD11b + F4/80 monocytes upon in vitro stretch of stromal cells. The migration of splenocytes to conditioned supernatants of stretched vs. non-stretched cells were tested by in vitro chemotaxis and measured by flow cytometry, migration index is shown with *n* = 8 independent samples from five independent experiments, MWU. **c** Increased accumulation of classical monocytes at mechanostressed tissues by biomechanical loading in arthritic disease. TNF^∆ARE^ mice were divided into three groups (score matched) with different loading regimens during 2 weeks: voluntary running, unloading or control. Control littermates were used as a negative control. Knee synovium, Achilles tendon, and spleen were isolated and prepared for flow cytometry. Increases of classical monocytes in response to loading were seen in synovial tissue, Achilles tendon but not spleen. Synovium and tendon 3 mice/group, spleen individual mice; seven independent experiments. Repeated measures one-way Anova *p* < 0.01, post-test Tukey’s Multiple comparison (respectively four (synovium) and three (tendon) groups were compared due to missing value in unloaded group). **d** Representative dot plot of control (top panel) and voluntary running (lower panel) mice show increased frequency of classical monocytes to loading in TNF^∆ARE^ synovium. **e** Human Achilles tendon of patients with chronic tendinopathy (heightened biomechanical stress) (*n* = 7) vs. patients unable to walk because of general spasticity (represents unloaded conditions) (*n* = 3), MWU. **f** Representative dot plot of isolated mononuclear cells of patients unable to walk because of general spasticity (upper panel) and patients with chronic tendinopathy (lower panel). A marked increase in classical monocytes was observed under conditions of increased mechanical strain. **g**, **h** Clinical scoring data of CCL2−/− mice and corresponding C57BL/6 littermates show a significant increase in arthritis severity by voluntary running in the C57BL/6 mice but not in CCL2−/− mice. MMRM day 3–10; *n* = 10/group. Error bars show the mean ± s.e.m., *p < 0.05, **p < 0.01, ***p < 0.001

Because the overall gene signatures pointed to active myeloid recruitment and mesenchymal tissue response, we focused on the putative crosstalk between stromal cells and myeloid cells. Synovial and Achilles tendon fibroblasts were isolated from C57BL/6 and TNF^∆ARE^ mice. Fibroblasts were subjected to a chronic stretch regimen in vitro using a bioreactor and supernatants were collected and analysed for chemokine release and MMP3 and IL-6 production. A marked increase of CCL2, CXCL1, MMP3, and IL6 expression was noted in fibroblasts exposed to mechanical stress (Fig. 6a).

To test the hypothesis that application of stretch on stromal cells was sufficient to result in myeloid cell recruitment we performed in vitro chemotaxis assays and analysed the subset of migrated myeloid cells in response to stretched fibroblasts. Using conditioned supernatants of stretched stromal cells of both C57BL/6 and TNF^∆ARE^ mice we found that stretch induced migration of classical inflammatory monocytes and F4/80+ macrophages, while no differences could be observed for the migration of T-cells, NK cells, neutrophils or myeloid dendritic cells (Fig. 6b). Taken together these experiments show that in vitro biomechanical forces cause fibroblasts to secrete CCL2 among other chemokines, responsible for the attraction of inflammatory monocytes towards the biomechanically-exposed sites. We next evaluated whether this recruitment of classical monocytes was also apparent in vivo. Therefore, we investigated accumulation of classical monocytes in synovium, tendon or spleen in response to a 2-week exposure to voluntary running, unloading or control conditions in TNF^∆ARE^ mice. After voluntary running a significant increase of classical monocytes in synovium and tendons was noted while no changes were observed in the spleen. Importantly no differences were noted to other non-classical monocytes or T cells, NK cells, neutrophils or myeloid dendritic cells (Fig. 6c, d, Supplementary Fig. 3a). We could extend these observations to human entheses by comparing myeloid composition in the Achilles tendon of patients with chronic tendinopathy (heightened biomechanical stress) and patients that were unable to walk because of general spasticity (represents unloaded conditions). The latter group received a surgical release of tendinopathy to facilitate rehabilitation. A striking increase was also noted in the number of classical monocytes in the Achilles tendon in the condition of increased biomechanical stress (Fig. 6e, f, Supplementary Fig. 3b).

**Fig. 7:**
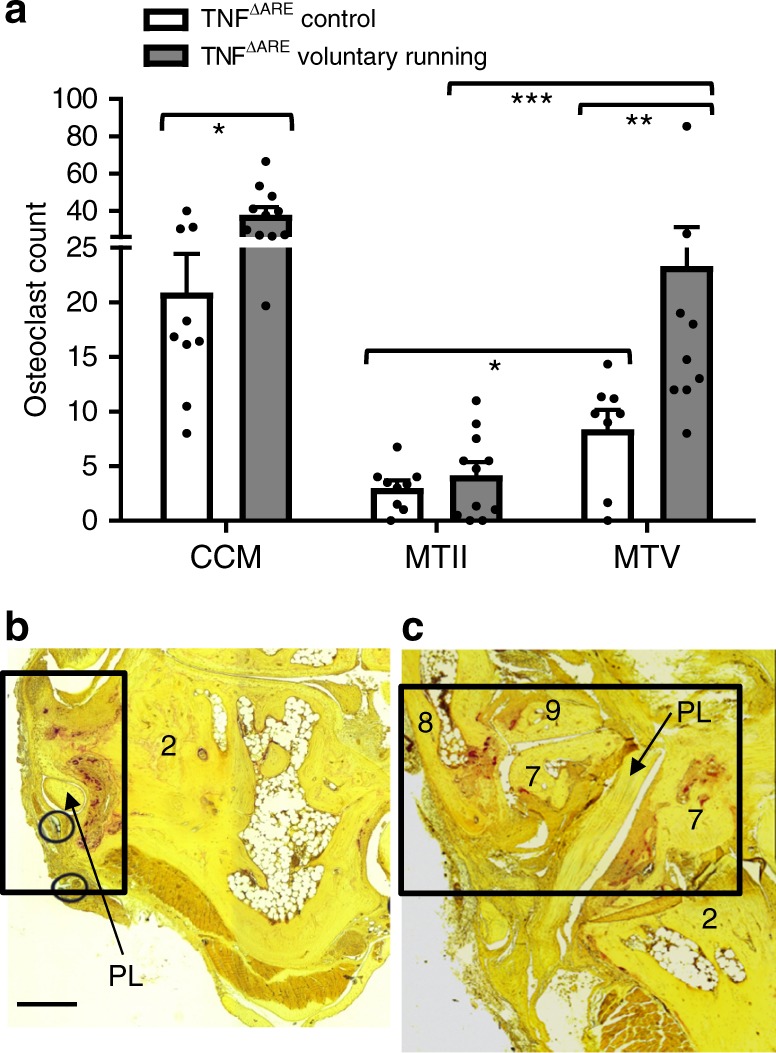
Increased osteoclast number in mechano-sensitive regions. TRAP staining of dorsal sections used for osteoclast count of the individual bones (minimal six sections, TNF^ΔARE^ Control (*n* = 9); TNF^ΔARE^ Voluntary running (*n* = 11) collected from two independent experiments. **a** Highest osteoclast numbers were noted at the CCM region, osteoclast number increases at this side with biomechanical stress (voluntary running compared to control condition). Significantly lower numbers of osteoclasts detectable in the enthesis-poor MTII vs. enthesis-rich MTV region; as well as parallel increase of osteoclast numbers with activity at the enthesis-rich MTV region, MWU. **b** The left panel shows in situ osteoclast localization at the peroneus groove; black rectangle = peroneus groove with peroneus longus (PL) at lateral side calcaneus. The right panel shows the (**c**) CM with localized osteoclast formation; black square encircles cuboid (plantar side) with peroneus longus running through the plantar groove where osteoclasts are localized, and the enthesis rich base of MTV. Scale bar: 0.25 mm. Error bars show the mean ± s.e.m., *p < 0.05, **p < 0.01, ***p < 0.001

To validate the in vivo effect of classical monocytes recruitment by voluntary running during arthritis development, a CAIA experiment was performed in CCL2−/− mice vs. wild-type controls. In the absence of CCL2, no increase in arthritis severity could be observed under voluntary running conditions. This is in striking contrast with wild-type control mice (Fig. 6g, h). This was also confirmed on histopathology (Supplementary Figure 2a). We also assessed the impact of pharmacological inhibition of CCR2 using neutralizing antibodies vs. isotype controls in C57BL/6 mice, clinically and by histopathology. These results indicate that CCR2 blockade abolished the effect of voluntary running on disease onset (Supplementary Figure 2b,c,d). Because monocytes can differentiate into bone resorbing osteoclasts under inflammatory conditions, we examined the effect of mechanical loading on frequency and distribution of osteoclasts in TNF^∆ARE^ mice by TRAP staining. A marked increase in osteoclast frequency was observed in voluntary running vs. control conditions (Fig. 7a). This was particularly apparent in mechanosensitive regions (CCM and MTV) vs. control regions such as MTII (Fig. 7a, b). These data mirror astonishingly well the bone erosion pattern highlighted by micro-CT (Fig. 3d). Collectively, our data demonstrate that mechanical strain in tissue resident stromal cells favors monocyte cell recruitment, as well as the differentiation of these cells into osteoclasts.

## Discussion

This study provides the first evidence that mechanical strain is an essential checkpoint for the translation of systemic immune activation into site-specific joint inflammation. The mechanism involved appears to be markedly conserved across several forms of murine and human arthritis. Our data show that mechanical strain is a key determinant for the onset of inflammation in mice, which are susceptible for arthritis, either due to arthritis-related induction of autoimmunity or due to genetically-induced cytokine dysbalance. Homing of inflammation to specific mechano-sensitive regions represents a novel paradigm and explains why arthritis in mice and humans is characterized by a regional and patchy distribution.

We have identified specific mechano-sensitive areas in the musculoskeletal system, such as the CCM region and the Cuneiform I-MTI junction, which are conserved primary homing places for arthritis. These regions are characterized by a high number of attachment sites and contact points for tendons and are therefore most influenced by biomechanical stress that is caused by bending and friction in addition to stretching of tendons, muscles or ligaments^14^. Notably, it is estimated that 70% of the tension exerted on bone depends on muscle contraction and tendon insertions, which illustrates the mechanical forces these sites are exposed to^15^. Our results also fortify epidemiological data suggesting a link between degree of physical activity and disease progression as well as onset of arthritis in humans and provide an anatomical and mechanistic basis for this association^16–18^.

Very few studies have addressed the pathways, which are involved in triggering local inflammation in arthritis. Some progress has been made in autoimmune arthritis explaining the transition from asymptomatic autoimmunity to inflammation and the homing of systemic autoimmunity to the joints^19^. Hence, glycosylation pattern of autoantibodies has shown to be crucial for their inflammatory properties and their potential to shift asymptomatic autoimmunity to inflammation. IL-23 essentially controls this glycosylation profile and promotes the pro-inflammatory properties of antibodies by inducing the cleavage of glycans from newly formed antibodies. While this mechanism explains the creation of a pro-inflammatory environment the reason for the homing of inflammation to the joints is not entirely explained by IL-23 suggesting an additional role for mechanical forces to be in place. Also mechanical strain did not seem to impact sialylation and galactosylation levels of autoantibodies. Other studies have shown that anti-citrullinated protein antibodies, the hallmark of autoimmunity in RA, bind and activate osteoclasts representing a kind of homing mechanism of inflammation to the joints, where osteoclasts are abundantly present^20^. While this mechanism may explain the homing of autoimmune-triggered arthritis to the joints, it still does not answer the question why some joint regions are more heavily involved by arthritis than others. Also, data that convincingly showed that pro-arthritic cytokines such as TNFα can activate resident mesenchymal cells and trigger arthritis, albeit explaining the mechanism of arthritis, did not answer the question why arthritis develops at certain predilection sites^21^. Our data close this knowledge gap and provide an attractive new concept explaining site specificity of arthritis.

While in mice the anatomical pattern of arthritis often seems remarkably conserved, these patterns vary substantially in humans. Such differences may be explained by higher heterogeneity of mechanical strain humans are exposed to, while mice face rather homogeneous conditions in a laboratory environment. Furthermore, different forms of arthritis in humans can be distinguished by their cytokine dependency and associated affection of different structures in the musculoskeletal systems. Hence, RA affects the synovial membranes (synovitis), while in SpA primarily entheseal structures (enthesitis) are involved. Mechanical factors have been recognized previously as the potential underlying determinants for the topographic pattern of articular changes in arthritis^4^. Our experiments do not mimic the differences between human RA and human SpA; however they suggest that overreaching principles exist, which link biomechanics to inflammation and tissue localisation. Herein, we show developmentally conserved mechanisms which link mechanical strain with biological responses preventing the over-use of the joints. Thus areas where a marked proximity of synovial and entheseal tissue is found (synovioentheseal complex) may resemble the highly conserved primary sites, where arthritis is homing to^12^.

This article shows that deleting *CCL2* or neutralizing *CCR2* is sufficient to diminish the effect of mechanical strain on the onset of arthritis, showing that the link between mechanical strain and the onset of arthritis appears to depend on the local recruitment of Ly6^high^ inflammatory monocytes elicited by mechanostress-induced chemokine induction such as CCL2 in resident mesenchymal cells. This observation was consolidated in human tissue samples, where clear differences were noted in the numbers of inflammatory monocytes in the presence or absence of loading. Other cells besides these inflammatory monocytes may have additional effects, like NK cells, innate lymphoid cells which are known to show increased activity during with physical activity. Furthermore, Ly6^high^ inflammatory monocytes represent the major precursor cells of osteoclasts in the inflamed joints^22,23^. Mechanically stimulated stromal cells could favor the differentiation into osteoclasts, which explains the high frequencies of bone erosions at biomechanical stressed areas. In line with this, induction of cathepsin K, an osteoclast specific gene^24^, was significantly induced by mechanical strain. This was mirrored by a strong increase in osteoclast numbers in mechanosensitive regions in the context of arthritis. Our data also indicate that intact adaptive immunity is not necessary in linking mechanical load with inflammation, since also T/B cell-deficient RAG deficient mice developed inflammatory responses under voluntary running conditions. However, this observation does not mean that adaptive immunity has no role in this process, i.e., autoantibodies act as enhancers of arthritis, promoting the chronicity of the locally induced inflammatory responses.

In summary, these data provide a new link between biomechanics and inflammation that explains the homing of inflammation to specific sites of the musculoskeletal system in arthritis. We propose that mechanical strain is sensed by mesenchymal cells in highly specified mechano-sensitive regions in the joints. At these sites, biomechanical forces are then translated to biochemical signals, such as chemokines, which initiate local inflammation and bone destruction through site-directed influx of inflammatory monocytes. Overall these findings do not only explain the typical site-specific onset of arthritis in mice and humans but also have immediate consequences for the prevention of arthritis by specific exercise programs.

## Methods

### Mice arthritis experiments and biomechanical conditions

Male C57BL/6 mice of 9–10 weeks old (Envigo, Netherlands) were subjected to “collagen-induced arthritis” (CIA). These mice were immunized intradermally (at the base of the tail) with 200 µg of chicken type II collagen (CII) (Morwell Diagnostics GmbH, Zurich, Switzerland) (in 0.1 M acetic acid) emulsified in Incomplete Freund’s Adjuvant + mycobacterium Tuberculosis H37RA (150 µg/mouse) (Difco, Lawrence, KS, USA) which forms complete Freund’s Adjuvant. After 21 days these C57BL/6 mice were again challenged (boost) with an injection of CII emulsified in complete Freund’s Adjuvant^25^. Mice were kept in three different biomechanical loading conditions for 4 weeks, starting 1 day after the second administration (boost; day 22). The first group consisted of CIA unloaded mice that were tail suspended to prevent loading of hind limbs (CIA unloaded mice). The second group contained mice with free access to a running wheel. The third group was kept in normal cages without running wheel (12 mice per group, two independent experiments). In a separate series of experiments additional groups were added. One group was unloaded at a later time point (day 26; 4 days after boost); another group contained mice in which voluntary running conditions were initiated 1 day after primary immunisation for CIA. TNF^∆ARE^ mice, age 6–9 weeks, were used for separate experiments^26^. Mice were kept in different biomechanical loading conditions (voluntary running vs. control (and unloading)) as described above. RAG2−/− mice (C57BL/6 background, male and female), age 9–10 weeks, were subjected to anti-collagen antibody induced arthritis (CAIA) by passive transfer of anti-collagen antibodies (Chondrex Inc., Androgen-CIA® 5-Clone Cocktail kit,4 mg/20 g mouse) in the presence or absence of LPS (injection of 5 µg at day3) (at least 6–12 mice/group; three independent experiments). C57BL/6 mice, age 10–12 weeks (Envigo, Netherlands), were subjected to CAIA (mdbiosciences, ArthritoMap^TM^ Antibody Cocktail, 2 mg/20 g mouse) with injection of 100 µg LPS at day 3 (five mice/group; three independent experiments). CCL2−/− mice (Jackson B6 129S4-CCL2^tm1Rol^/J, male) and corresponding C57BL/6J controls (Charles River, male), age 9–10 weeks were subjected to CAIA (mdbiosciences, ArthritoMap^TM^ Antibody Cocktail, 2 mg/20 g mouse) with injection of 100 µg LPS at day 3 (10 mice/group). Ten-week old C57BL/6 (Envigo, male) mice were subjected to either CCR2 antibody (MC-21)^27^ or isotype (MC-67) (both generously given by Prof. Matthias Mack) by IP injection (both 15 µg/20 g daily), followed by CAIA (mdbiosciences, ArthritoMap^TM^ Antibody Cocktail, 2 mg/20 g mouse) induction after 6 h and by injection of 100 µg LPS at day 2 (10 mice/group). Sample size of the mice experiments was decided based on disease incidence of previous experiments and with G-power analyses using means and standard deviation of previous experiments. In the C57BL/6 and CCL2−/− CIA/CAIA mice experiments the equal age male mice were divided randomly on base of weight and initial cage number in the three different conditions (voluntary running, control, and unloading). CAIA RAG2−/− mice were equally divides between the three conditions, based on sex, weight and age. The transgenic TNF^∆ARE^ mice were similar age and sex and were scored clinically before start of the experiment to be able to divide them equally between the three conditions. Housing was performed according to institutional guidelines and all experiments were conducted according to guidelines and approved by the Ethics Committee of Laboratory Animal Welfare of Ghent University.

### CIA, CAIA, and TNF^∆ARE^ clinical arthritis scoring

CIA paws were monitored for clinical symptoms each other day till the day of sacrifice (day 51). Disease severity scoring system was done as previously described^25^. Clinical evaluation was performed by two investigators and mean of both scores was calculated. CAIA paws were monitored clinically each day till day of sacrifice (day 14); the same severity scoring system was used as with CIA.

For the TNF^∆ARE^ experiment the paws were monitored for clinical symptoms at the start and further on every week during the 2–4 weeks experiment. Disease severity scoring system: 0 = normal; 1 = distortion of foot morphology; 2 = swelling at foot; 3 = deformation (and swelling) foot and toes. Clinical scoring was done respectively semi-blinded and full blinded by two experienced investigators (minimal differences observed between both), all further sample analyses like H&E, bone erosion and ELISA was done blinded.

### Histology

Paws, knees, and toes were fixed in 4% formaldehyde, decalcified with formic acid and embedded in paraffin. Serial sections of the paws, knees, and toes were stained with haematoxylin and eosin (H&E) or with safranin O-fast green. These sections were scored for inflammation and joint damage by two independent assessors and mean scores were calculated. In the first series of experiments inflammation was scored globally (foot, ankle, and SEC) as a sum of excudate (number of inflammatory cells in the synovial cavity) and infiltrate (numbers of inflammatory cells in the synovial tissue). Scoring range goes from 0 (no inflammation) to 3 (severe inflamed joint). Additionally, erosion was scored globally on base of cortex and cartilage destruction ranging from 0 (no erosions) to 3: 1 = cortex is eroded superficially at a few places, non to little cartilage damage; 2 = cortex is eroded fully reaching bone marrow, contour articular cartilage damaged; 3 = completely eroded cortex with cells coming into bone marrow, severe loss of articular cartilage. Further, in the second series of experiments (only CIA C57BL/6) paws were fully sectioned and stained to be able to map the detailed inflammation pattern of the entire foot of the mice, subjected to different biomechanical conditions. The scoring system was adapted as follows: inflammation at independent joints between individual bones was registered for hindfoot, midfoot, and the proximal part of the forefoot.

### Anti-collagen antibody levels and glycosylation pattern

Blocking was performed with 0.1% casein with 1 h incubation time at (4 °C). Serum was used from mice before second boost (day 21) and at the end of the experiment. Serum was used at dilution 1/100 and incubation was done overnight. For detection antibodies IgG1-HRP and IgG2-HRP were used in a 1/4000 dilution with 1 h incubation at RT. To determine the IgG Fc glycan galactosylation and sialylation, we specifically released IgG Fc glycans in 4 µl serum by hydrolysis with 40 units of endoS (IgGZERO, Genovis, Sweden) for 1 h at 37 °C.

For calculating relative sialylation levels, the released N-glycans were labeled with 8-aminopyrene-1,3,6-trisulfonic acid (APTS) and analyzed on an ABI3130 DNA sequencer^28^. Peak heights for the seven main glycan peaks were measured. These peaks are G0F (asialo, agalacto core fucosylated biantennary N-glycan), G1F (2 isomers: G1F_1 and G1F_2, asialo, monogalacto, core fucosylated biantennary N-glycan), G2F (asialo, digalacto, core fucosylated biantennary N-glycan), G1FS1 (2 isomers: G1FS1_1 and G1FS1_2, core fucosylated biantennary N-glycan with 1 galactose and sialic acid on either of the 2 antennae) and the G2FS1 peak (monosialo, biantennary N-glycan). The relative sialylation levels were calculated as:

$$({\mathrm{G2FS1}} + {\mathrm{G1FS1}}\_1 + {\mathrm{G1FS1}}\_2)\,{\mathrm{per}}\,{\mathrm{total}}\,{\mathrm{peak}}\,{\mathrm{height}}$$

We need to note here that the calculated relative sialylation levels do not correspond to real sialylation percentages, since the analysis technique used (capillary electrophoresis with electrokinetic injection) overestimates analytes with extra negative charges (e.g., sialic acid residues) due to preferential injection of those analytes over uncharged analytes.

For calculating undergalactosylation scores (UGS), the released N-glycans were labeled with APTS, digested with *A. ureafaciens* neuraminidase (in house production) and analyzed on an ABI3130 DNA sequencer^28^. Peak heights for the 4 glycan peaks; G0F, G1F_1, G1F_2 and G2F were measured and the UGS were calculated as:

$$\left( {\mathrm{G0F}} + 0.5 \ast {\mathrm{G1F}}\_1 + 0.5 \ast {\mathrm{G1F}}\_2 \right) \ast \left( {\mathrm{G0F}} + {\mathrm{G1F}}\_1 + {\mathrm{G1F}}\_2 + {\mathrm{G2F}} \right) \wedge - 1$$

### Micro-CT acquisitions

Micro-CT scans of samples (*n* = 75) were performed at the Center for X-ray Tomography of the Ghent University (UGCT, www.ugct.ugent.be). The required voxel size defined the dimensions of the scanning region which included full hindfoot and midfoot and part of forefoot. The first samples were scanned with a micro CT scanner with a Feinfocus transmission tube and a Varian Paxscan detector with a pixel pitch of 127 µm (tube voltage 100 kV, tube power 9 W, no filtration)^29^. For every scan 1800 projections were taken and the exposure time of every projection was 1 s. The resulting scan images have a voxel size of 4.5 µm. The second batch of samples were scanned on HECTOR (developed in collaboration with the Ghent University spin-off company XRE, www.xre.be)^30^, using a directional X-ray source set at 130 kV and 10 W target power with a 1 mm Aluminum filtration. The detector was a Perkin-Elmer flat panel measuring 40 × 40 cm, with a pixel pitch of 200 µm. A total of 2000 projections of 1 s exposure time each were recorded. The resulting scan images have a voxel size of 4 µm. The obtained projection images of all scans were reconstructed using Octopus Reconstruction^31^, a commercial software licensed by XRE and originally developed by UGCT, based on the standard FDK-algorithm for reconstruction of CT data. 3D visualisations were made using the commercial rendering software VGStudioMAX (Volume Graphics).

### Bone erosion quantification

Bone erosion on micro-CT scans was calculated on reconstructed data with an in-house written Fiji script. The program determines the bone surface and bone inner space and fills the pores in the bone surface. The difference between the filled surface and the normal non-filled bone surface was calculated, dividing this difference by the normal bone data gives the erosion %. Additionally, an extended version of this program gives the opportunity to determine quantitative data for individual bones or even certain parts of a bone. This extended version gives additional parameters such as quantification of the pores present in the cortex (full pores in contrast with erosion %, where erosion is only considered over a defined distance of the surface). Data on inflammation associated bone erosion were conducted with mice that had a clinical score of ≥1 on the scanned hind paw under various conditions (not applicable for CIA unloaded), as pre-established. Bone erosion data were normalized by wild-type mice data taken within the same micro-CT experiment. Mice of comparable clinical score were used to compare bone erosion between CIA control and voluntary running as well as TNF^∆ARE^ control and voluntary running mice.

### Anatomical study

A microanatomy study was conducted by combining data collected from detailed anatomical dissections of 50 mice, combined with H&E full sections and data from micro-CT scans. For the anatomical study a Zeiss stereo microscope Discovery V12 was used and conducted together with an expert morphology (P.S.). Anatomical data were compared and named according to existing nomenclature^32,33^. Human nomenclature was used for consistency with human data. Anatomical figures were made on base of our anatomical study and micro-CT scans by BixPixel (www.bigpixel.nl, Jeroen Hoekstra and Ferdinand Assenberg) using Autodesk Maya and Unity.

### X-ray and 3D micro-CT in human RA and SpA patients

X-rays of the feet were collected from a consecutive cohort of 63 RA patients (19 male, 44 female; mean age 59.3 ± 11.55). Radiographic damage was assessed by a modified Sharp-van der Heijde scoring by two independent assessors. The joints were typically divided in four quadrants. Erosion score ranged between 1 and 5. A score of 1 represents erosion only in 1 quadrants, a score of 4 was given when all quadrants were affected by erosion. A maximal score of 5 was given if the joint was entirely destructed. Narrowing of the joint space was also scored by a 1 when present and 0 when absent. All patients gave informed consent and the ethical committee of the University Hospital of Ghent granted permission to perform this study.

High-resolution peripheral quantitative computed tomography (HR-pQCT) imaging of the right foot was performed in five healthy controls and ten age-matched and gender-matched SpA patients (fulfilling ASAS classification criteria). For scanning an Xtreme CT device (Scanco, Bruettisellen, Switzerland) was used. Scans were (i) performed at a line connecting the first cuneiform bone with the cuboid bone in the midfoot and (ii) at the Achilles tendon insertion according to the manufacturer’s standard protocol. The foot was immobilized using a carbon fiber shell to reduce movement. Standardization of measurements was insured by daily cross-calibrations with a standardized control phantom (Moehrendorf, Germany). The scan regions of interest were examined in 110 parallel slices (82 μm voxel size) with a total measurement time of 2.8 min. All measurements and evaluations were performed using the manufacturer’s standard software. Entheseophyte size (maximal height in mm over cortical bone surface) was analyzed at three different mechanically exposed sites: (i) lateral side of the cuboid bone, (ii) medial side of the first cuneiform bone, and (ii) Achilles insertion side. The study was conducted upon approval of the local ethic committee of the University of Erlangen, each patient provided informed consent.

### Gene expression in Achilles tendon and human synovial tissue

Achilles tendons (inclusive enthesis) were dissected from C57BL/6 (Envigo) mice subjected to three different loading conditions (voluntary running (6), or control (3)) for 14 days. Isolated tendons were stored in RNAlater® (Qiagen) before RNA isolation with microRNeasy Kit (Qiagen). Microarray assays, quality controls, and statistics were successfully done by Nucleomics core VIB (Leuven) with Chip Mouse 430_2.0. The CEL files generated by these arrays are analysed by Affymetrix Expression Console^TM^ Software. Normalized expression values of the different conditions were compared with Limma package of Bioconductor. The resulting *p*-values are corrected for multiple testing with Benjamini-Hochberg to control the false discovery rate. Microarray data will be deposited in a public repository before publication.

Achilles tendons were dissected from TNF^∆ARE^ mice subjected to three different loading conditions (voluntary running or control) for 14 days. Isolated tendons were stored in RNAlater® (Qiagen) before RNA isolation with microRNeasy Kit (Qiagen). Preamplification was used to increase cDNA yield (QuantiTect Whole Transcriptome kit, Qiagen). Real-time PCR was performed on a LightCycler 480 (Roch Molecular Diagnostics) using SYBR-green Mastermix (SensiMix SYBR No Rox-kit, Bioline). Expression values were normalized to TBP, B2M, GCC1, and HPRT. Used primers BIORAD (PrimePCR assays) have a unique assay ID number: *CCL2* (ENSMUSG00000035385, qMmuCED0048300), *ICAM1* (ENSMUSG00000037405, qHsaCED0004281), *LILRA6* (ENSMUSG00000030427, qMmuCED0026189), *CTSK* (ENSMUSG00000028111, qMmuCID0022824), *MMP3* (ENSMUSG00000043613, qMmuCED0049170), *LOXL2* (ENSMUSG00000034205, qMmuCED0061099), *THY1* (ENSMUSG00000032011, qMmuCED0047945), *LUM* (ENSMUSG00000036446, qMmuCED0048548), *CFP* (ENSMUSG00000001128, qMmuCID0022409), *C1QTNF6* (ENSMUSG00000022440, qMmuCED0039651), *CCL8* (ENSMUSG00000009185, qMmuCED0045281), *TNC* (ENSMUSG00000028364, qMmuCED0047470), *NFATC1* (ENSMUSG00000033016, qMmuCID0040129), *BMP1* (ENSMUSG00000022098, qMmuCED0045542) and *TBP* (ENSMUSG00000014767, qMmuCID0040542), *GCC1* (ENSMUSG00000029708, qMmuCED0046833), *PIGO* (ENSMUSG00000028454, qMmuCID0006969). Used mouse primers QIAGEN (QiantiTect Primer assays) with following catalogues number: *HPRT* (ENSMUSG00000025630, Mm_Hprt_1_SG, QT00166768) *TBP* (ENSMUSG00000014767, Mm_Tbp_1_SG, QT00198443). Used mouse primers INVITROGEN (Custom DNA oligos): *IL6* (ENSMUSG00000025746, IL6 Reverse TGTGAAGTAGGGAAGGCCGTGGT, IL6 Forward TGCAAGAGACTTCCATCCAGTTGCC), *CXCL1* (ENSMUSG00000029380, CXCL1 Reverse GTGCCATCAGAGCAGTCTGT, CXCL1 Forward ACTCAAGAATGGTCGCGAGG), *CCR2* (ENSMUSG00000049103, CCR2 Reverse TCATACGGTGTGGTGGCCCCT, CCR2 Forward GGGCATTGGATTCACCACATGTGC).

Human synovial tissue samples were gathered by the group of Rik Lories (KULeuven). *n* = 4, mean age 55.25 ± 14.5 (female/male 3/1, RF+/RF− 1/3, ACPA+/ACPA− 2/2). No DMARD or biological on the moment of biopsy. All patients gave informed consent and the ethical committee of the University Hospital of Leuven granted permission to perform this study. Used human primers BIORAD (PrimePCR assays) have a unique assay ID number and used reference genes are TBP, GCC1, and PIGO. *CCL2* (ENSG00000108691, qHsaCID0011608), *ICAM1* (ENSG00000090339, qHsaCED0004281), *LILRA6* (ENSG00000204577, qHsaCED0005804), *CTSK* (ENSG00000143387, qHsaCID0016934), *MMP3* (ENSG00000149968, qHsaCID0006170), *LOXL2* (ENSG00000134013, qHsaCED0044522), *THY1* (ENSG00000154096, qHsaCED0036661), *LUM* (ENSG00000139329, qHsaCID0015376), *CFP* (ENSG00000126759, qHsaCED0002165), *C1QTNF6* (ENSG00000133466, qHsaCED0001807), *CCL8* (ENSG00000108700, qHsaCED0043240), *TNC* (ENSG00000041982, qHsaCED0057376), *NFATC1* (ENSG00000131196, qHsaCED0044370), *BMP1* (ENSG00000168487, qHsaCID0010875) and *TBP* (ENSG00000112592, qHsaCID0007122), *GCC1* (ENSG00000179562, qHsaCID0014581), *PIGO* (ENSG00000165282, qHsaCID0010709).

Ingenuity Pathway Analyses (IPA; Ingenuity Systems, Inc., Redwood City, CA, USA) were used to examine canonical pathways and biological networks associated with the differential expressed genes. The treemap visualization was done using the R ‘portfolio’ package (https://github.com/dgerlanc/portfolio) and is based on the prior calculation of the activation *z*-scores, which infer the activation states biological functions and pathways.

### In vitro stretch of primary fibroblasts

Synovium and Achilles tendon (including the enthesis) of individual mice were isolated with a stereo microscope (Zeiss stereo microscope discovery V12) and transferred into 24 well plates and cultured till confluent in T75 plates. Cells were seeded 50,000 per 6 well on a flexible membrane (BioFlex^**®**^ Culture Plates, Flexcell international corporation). After 48 h of incubation at 37 °C, wells were either stretched or not. A chronic type of stretch protocol (mimicking physical activities like walking) was applied: square wave (mimic load curve during walking), a frequency of 0.5 Hz, elongation of 6%, and a time schedule of 30′ of stretch followed by 10′ of rest, repeated twice. Supernatants of (none) stretched fibroblasts were screened for CCL2 (eBioscience), IL6 (eBioscience), MMP3 (R&Dsystems), and CXCL1(R&Dsystems) concentration by ELISA.

### In vitro chemotaxis

Chemotaxis experiments were conducted as previously described^34^. Briefly, transwells were used (3 µm pores; Corning Inc) and the migration of splenocytes (1 × 10^6^ cells) from CB57BL/6 litter mate control mice was assessed after 3 h incubation using supernatants from stretched vs. non-stretched primary fibroblasts. The in vitro migrated cells were characterized by multicolor flow cytometry (FACS CANTO, BD Pharmingen) using antibodies against CD3e-V500 (BD Pharmingen, 5600773; 1/50), NK1.1 (PEcy7, 25-5941-82; 1/60), CD11b (PercPcy5.5, 45-0112-80; 1/60), CD11c (PE, 12-0114-83; 1/50), Ly6g (APCcy7, 560602; 1/120), Ly6c (APC, 17–5932; 1/240), F4/80 (FITC, 11-4801-82, 1/60) (all from eBioscience). Fluorescent beads (2 mu, Polysciences Inc.) were added as internal control. Statistical calculation was done on eight independent samples. Migration index was calculated as:$\left( {\frac{{{\mathrm{migrated}}\,{\mathrm{cells}}\,{\mathrm{sample}}}}{{\mathrm{beads}}}} \right) \ast \left( {\frac{{\mathrm{migrated}\,{\mathrm{cells}}\,{\mathrm{control}}}}{{\mathrm{beads}}}} \right)^{ - 1}$.

### In vivo immune phenotype response to mechanical strain

Male TNF^∆ARE^ mice, age 8–10 weeks, were assigned to a control, voluntary running or unloaded regimen (3–4 mice per group) for 2 weeks, mice were corrected for age and clinical scoring before onset of the conditions. Knee synovium, Achilles tendon (including enthesis) and spleen were collected in RPMI medium. Synovium and tendon were subjected to an enzymatic digestion with collagenase D (Sigma Aldrich), and DispaseII (Gibco). Splenocytes were obtained by lympholyte-M isolation. All samples were then characterized for cell composition by multi-color flowcytometry using an identical panel used for in vitro chemotaxis experiments. Because of one missing sample (too limited cell yield) in the tendon TNF^∆ARE^ unloaded condition (Fig. 6c), it was not possible to include this condition in the one-way anova repeated measurements analysis without diminishing the sample size, this condition was not included in the analysis of TNF^∆ARE^ tendon samples.

Human samples were obtained from patients requiring surgery for chronic Achilles tendinopathy (*n* = 7, mean age 52.7 ± 2.95) and represented the overloading condition. Patients with a hemiplegia (*n* = 3, mean age 33.4 ± 20.7) who required a surgical Achilles tendon lengthening because of spasticity served as a control group by representing the underloading condition due to their immobility on the affected side. After surgical resection, samples were collected into complete culture medium (DMEM, Gibco, Life Technologies) and sliced into small pieces and digested by collagenase D and Dispase II. Immunophenotyping was determined by flow cytometry using anti-human fluorochrome-conjugated monoclonal antibodies (mAbs) with following panel: CD11b (FITC, 11-0118-41, 1/20), CD14 (Amcyan, 561392, 1/25), CD16 (PE, 12-0167-42, 1/25), CD3e (APC, 17-0038-41, 1/20), CD19 (APC-eF780, A15429, 1/50), CD90 (PEcy7, 17-0909-41, 1/20) (all from eBioscience), and CD45 (PerCP-Cy5.5, 67-9459-T100, 1/34). All patients gave informed consent and the ethical committee of the University Hospital of Ghent granted permission to perform the study. All analyses were performed using FlowJo software.

### TRAP staining

Paws were fixed in 4% formaldehyde, decalcified with Ethylenediaminetetraacetic acid (EDTA) and embedded in paraffin. Hind paws were dissected dorsally, at least six slides from each paw were stained with Tartrate-resistant acid phosphatase (TRAP) (Sigma-Aldrich). A 2 h staining was performed followed by a 2 min wash with H_2_O without the need of an extra hematoxylin staining.

### Statistical analysis

Statistical analyses were performed using SPSS, GraphPad Prism and GenStat. Experimental values are presented as mean ± SEM. Statistical differences were evaluated using the two-tailed unpaired non-parametric Mann–Whitney *U*-test (MWU). Larger samples were checked for normality distribution with Kolmogorov Smirnov and Shapiro–Wilk test (<0.001) and therefore analysed with a non-parametric (non-) paired (respectively Mann–Whitney, Wilcoxon matched pairs signed rank test). Data of groups were compared by the non-parametric Kruskal–Wallis test with post-test. Data of groups with normal distribution (Kolmogorov Smirnov) and repeated measurements were analyzed by one-way ANOVA repeated measurements followed by a post-test (Fig. 6c). Clinical scores were analyzed as repeated measurements using the residual maximum likelihood (REML) approach as implemented in Genstat v18 (mixed models repeated measurements, MMRM). Briefly, a linear mixed model with replicate, genotype, time, and treatment × time interaction as fixed terms, and subject.time used as residual term, was fitted to data. Times of measurement were set at equal intervals and the autoregressive correlation structure of order 1 (AR1) (for Fig. 1g) and uniform distribution (for Fig. 1b) were selected as best model fits based on the Akaike Information Coefficient. Significances of the fixed terms and significances of changes in differences between genotype effects over time were assessed by an *F*-test. In analogy with survival analysis, incidence data were defined as the time it takes for the arthritis to manifest itself (score ≥ 1 for at least two consecutive days) in the mouse. Typically these data are censored, i.e., some mice stay healthy beyond the end of the study, so their incidence time is unknown. The incidence function *F*(*t*) was defined as the probability that an individual was not affected by the arthritis at time *t*. Equality of incidence curves between groups was tested using the log-rank test. Significant differences between experimental groups were represented as *p < 0.05, **p < 0.01, ***p < 0.001.

## Electronic supplementary material

Supplementary Information

Description of Additional Supplementary Files

Supplementary Movie 1

Supplementary Movie 2

Supplementary Software 1

Reporting Summary

## Data Availability

Accession code for microarray depossed at GEO is GSE120027. The data that support the findings in this study are available from the corresponding author upon request.
