# Supplementary material for: Mechanical strain determines the site-specific localization of inflammation and tissue damage in arthritis
Source: Nat Commun. 2018 Nov 5;9:4613. doi: 10.1038/s41467-018-06933-4 (PMC6218475; doi:10.1038/s41467-018-06933-4)
Supplement: Supplementary file 1 — Supplementary Information [file 41467_2018_6933_MOESM1_ESM.pdf]

Supplementary Note 1: detailed anatomical description

The calcaneus-cuboid-MTV region contains four entheseal organs and three functional entheses (eg. sites where tendon wraps around a bone pulley and associated with a tendon sheath lined by a synovial membrane, rather than insertion in the immediate vincinity of these regions[11]. Two functional entheses are created by the peroneus longus (PL) tendons (details in Figure **3a2, 3b2**) at the lateral distal side of the calcaneus and at the lateral plantar side of the cuboid bone. At these locations two other entheseal organs are formed by the calcaneocuboid ligament at the lateroplantar side of the calcaneus and lateral side of the cuboid bone.

At the base of MTV, two entheseal organs are formed by the peroneus brevis (PB) and the abductor digit V tendon (AV). Additionally, a functional enthesis is formed by the extensor digitorum lateralis (DL) at the dorsolateral site of MTV. In contrast, MTII only has negligible entheses formed by the very small interosseous muscles. The entheseal organs at MTV, distal calcaneus and the cuboid bone are formed by large tendons (“key players”) (PL, PB) in stabilizing the foot upon every movement. As these tendons control mobility and strict position of the bones, these sites are particularly prone to repetitive large mechanical stress[14].

At cuneiform I, we mapped four entheseal organs and one functional enthesis. Here, the most important entheseal organ is formed by the attachment of the stabilizing PL tendon at the distal medioplantar side of cuneiform I. Another large tendon attaching medioplantar to the bone is tibialis anterior tendon (TA), which forms both an enthesis and a functional enthesis. Additionally, two entheseal organs and a functional enthesis are formed by three ligaments attaching to the cuneiform I bone (respectively distal ligament (dl), lateral ligament (ll), os sesamoideum tarsale-MTI ligament (oml)). These form entheseal hotspots. Similar to the CCM region, these tendons control mobility and strict position of the bones. Therefore these sites are also particularly prone to repetitive large mechanical stress

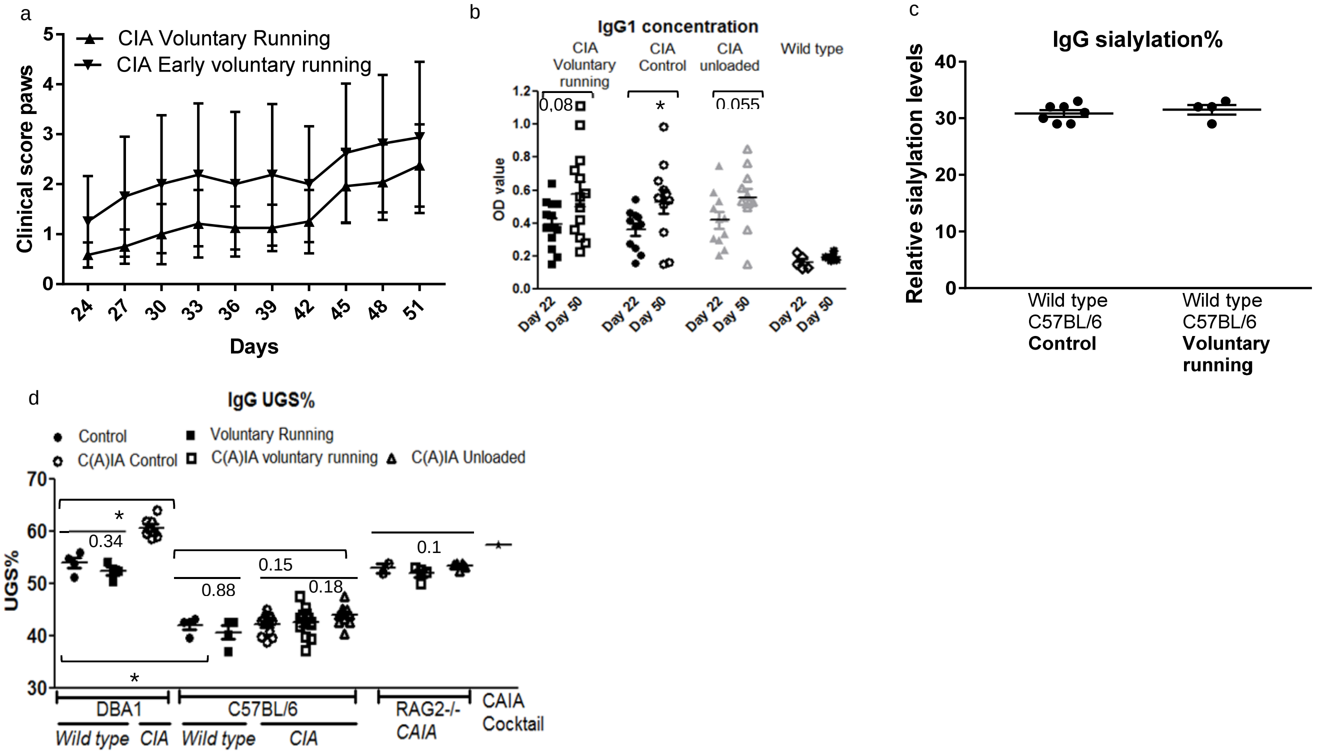

Supplementary Figure 1: Biomechanical conditions do not affect IgG production or glycosylation. a) Clinical scoring of paws from CIA mice that had been running from day 22 (voluntary running) or from day 1 (early voluntary running) after immunization. No differences could be observed between both regimes, respectively n=12 and n= 8, MMRM. **b)** Anti-collagen type 2 IgG1 levels were determined by ELISA in CIA under different loading conditions. Results indicate OD values at day 22 and at the end of the experiment (day 51) after primary immunization. In all groups the IgG1 levels are significantly increased throughout the experiment, n=13/group for CIA C57BL/6 mice and n=5/group for wild type mice C57BL/6 mice, MWU. **c)** Relative sialylation levels are shown from wild type C57BL/6 mice that had been in control (n=7) or voluntary running (n=4) condition during 14 days. No differences are seen between both conditions, MWU. **d)** Undergalactosylation% determined for wild type DBA/1 mice and wild type C57BL/6 mice in different biomechanical conditions (control, voluntary running) showing no effect of voluntary running on the galactosylation of IgG, n=4 per group, MWU. Additionally, data of CIA C57BL/6 show no differences in UGS% of IgG (self-produced antibodies) between the biomechanical conditions (control, voluntary running, tail suspension) at day 35 of the experiment in contrast with clear differences in clinical scoring (see Fig.1), n=10-13 each condition, KW. In line herewith no differences could be observed in UGS% of IgG (injected antibodies) between CAIA RAG2-/- mice subjected to different biomechanical conditions, n=2-5/group, KW. As reported in earlier papers, induction of CIA results in IgG undergalactosylation in DBA/1 mice, n=4 for wild type DBA/1 and n=8 for CIA DBA/1, MWU. As a positive control also UGS% of the CAIA cocktail is determined by this method. Error bars show the mean ± s.e.m.,* p<0.05, ** p<0.01.

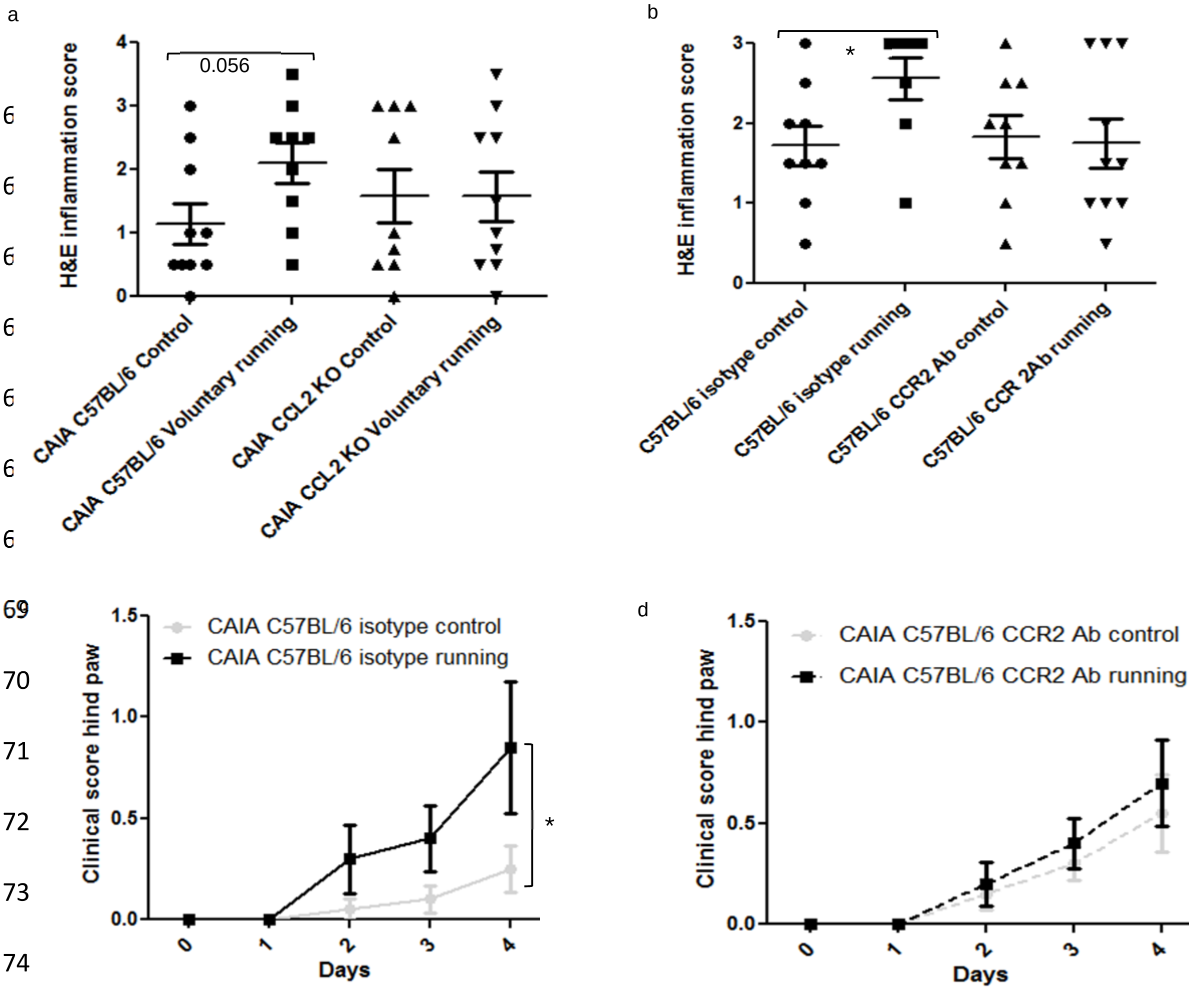

Supplementary Figure 2: In vivo search for a link between the CCL2-CCR2 axis and voluntary running. a) H&E inflammation score of CAIA C57BL/6 mice and CCL2-/- mice at day 14, showing an almost significant increase in inflammation by voluntary running in CAIA C57BL/6 mice, which is not present in the CCL2-/- mice, n=10 per condition, MWU. **b)** H&E inflammation score of CAIA C57BL/6 mice daily treated with CCR2 Ab or isotype (IP), showing a significant increase in inflammation by voluntary running in CAIA C57BL/6 isotype treated mice, which is not present in the CAIA C57BL/6 CCR2 Ab treated mice, n=10 per condition, MWU. **c)** Clinical scoring of CAIA C57BL/6 mice treated with CCR2Ab or isoytpe. Treatment and follow up was only possible during 5 day. By neutralizing CCR2 the effect of voluntary running on the onset of arthritis is diminished (lower panel), in contrast a significant increase in clinical scoring by voluntary running is seen in C57BL/6 mice treated with isotype (upper panel), n=10/group, MMRM day 1-4. Error bars show the mean ± s.e.m.,* $p<0.05$.

a

Gating strategy mouse tendons

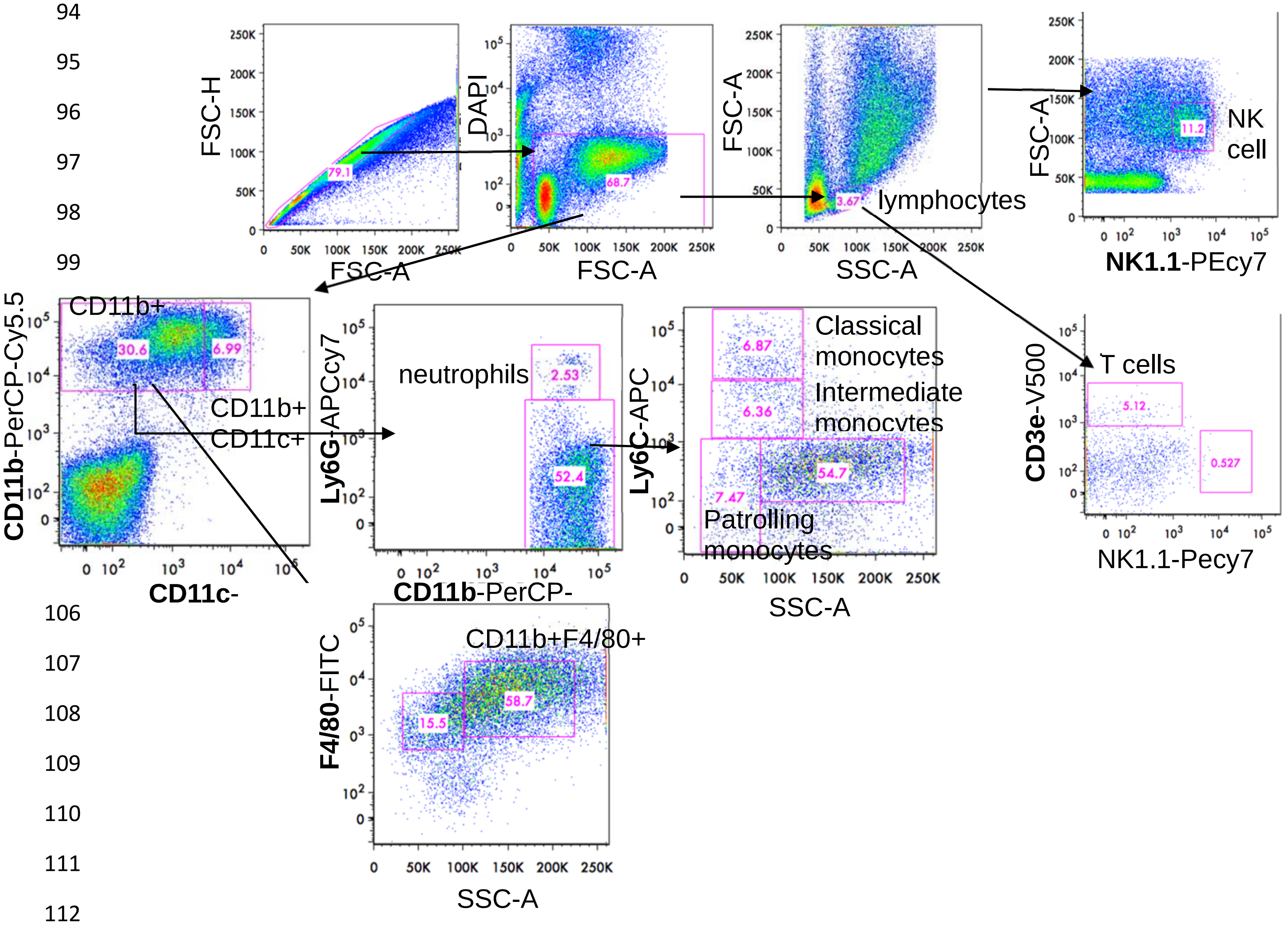

b

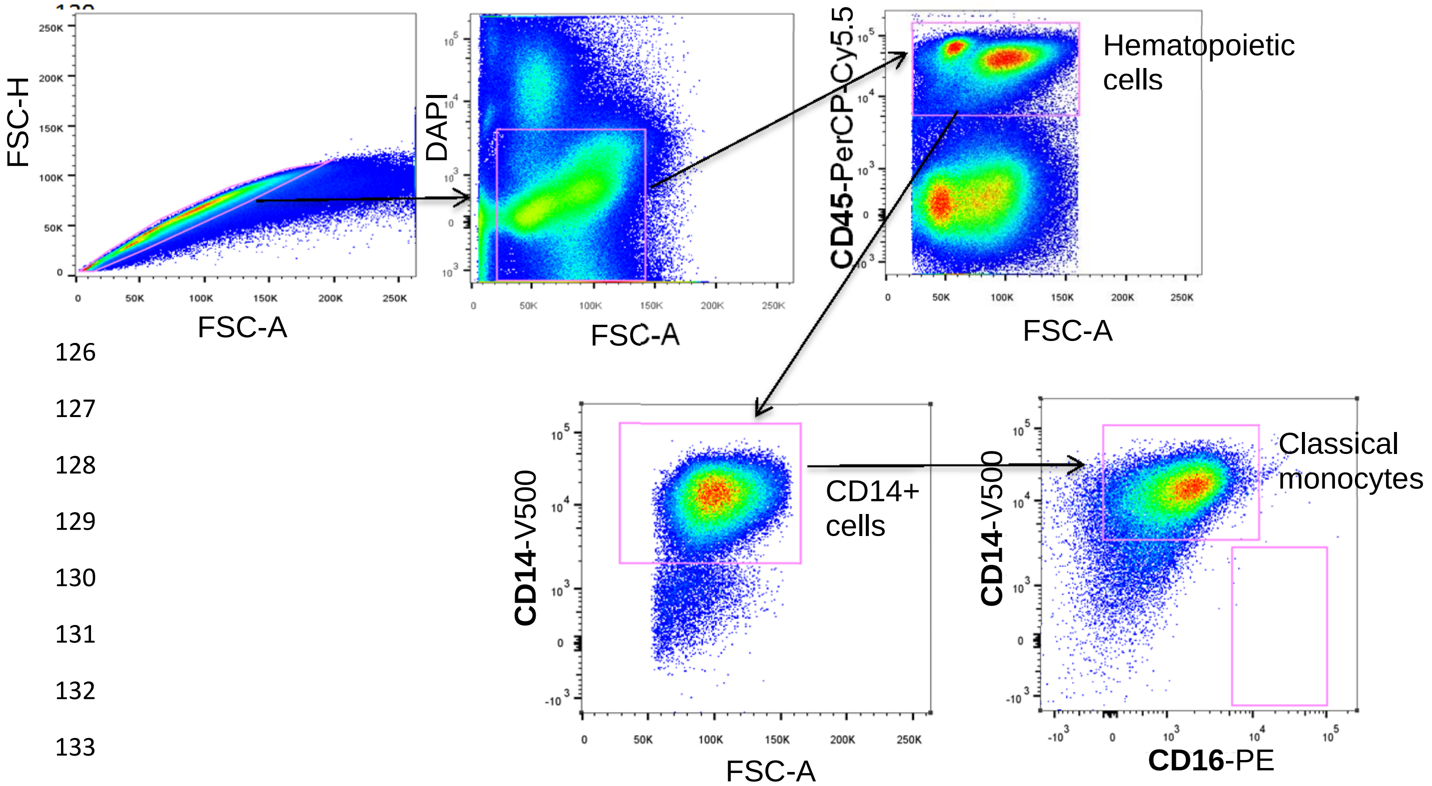

Supplementary Figure 3: Used gating strategies in mouse and human samples. a) Strategy for Figure 6d: First doublets were removed, then dead cells were excluded by use of DAPI, starting from those DAPI- singlet cells we determined NK cells by NK1.1 positive cells. In the lymphocyte gate we seperated T cells by use of CD3e and NK cells by use of NK1.1. Further gating was on all DAPI- cells except CD3e+ and NK1.1+ cells, these cells were gated by use of CD11b and CD11c markers. CD11b+ cells were subdivided by use of the F4/80 marker (identifying macrophages) or by the Ly6G marker identifying neutrophils. Ly6G- cells were further separated by use of Ly6C to identify classical monocytes (SSC-A low and LY6C ++), patrolling monocytes (SSC-A low and Ly6C low) and intermediate monotyes (SSC-A low Ly6C+). **b)** Strategy for Figure 6f: Gating was started by removing the doublet cells. The alive cells were gated from the DAPI negative cells and used for further analysis. The hematopoietic cell population was gated in the CD45+ population, leaving out the stromal cells. Out of this the CD11B population was selected. Out of this population the CD14++and CD16+ positive cells could be demonstrated.
