# Supplementary material for: Mechanical strain determines the site-specific localization of inflammation and tissue damage in arthritis
Source: Nat Commun. 2018 Nov 5;9:4613. doi: 10.1038/s41467-018-06933-4 (PMC6218475; doi:10.1038/s41467-018-06933-4)
Supplement: Supplementary file 2 — Description of Additional Supplementary Files [file 41467_2018_6933_MOESM2_ESM.pdf]

Description of Additional Supplementary Files

File Name: Supplementary Movie 1
Description: 3D reconstructions of the cuboid bone of CIA voluntary running mice which show clear erosions at mechano-stressed locations.

File Name: Supplementary Movie 2
Description: 3D reconstructions of the cuboid bone of C57BL/6 wild type mice showing no erosions.

File Name: Supplementary Software 1
Description: Interactive anatomical application. Tendons and muscles are shown on a micro-CT scan of a hindfoot (healthy mouse). Individual names can be seen by moving on the individual tendons/muscles. Individual bones and tendons can be removed (and added) by clicking on them.
