# Supplementary material for: Mechanical strain determines the site-specific localization of inflammation and tissue damage in arthritis
Source: Nat Commun. 2018 Nov 5;9:4613. doi: 10.1038/s41467-018-06933-4 (PMC6218475; doi:10.1038/s41467-018-06933-4)
Supplement: Supplementary file 6 — Reporting Summary [file 41467_2018_6933_MOESM6_ESM.pdf]

nature research

Corresponding author(s): DBPR NCOMMS-18-00421A

Reporting Summary

Nature Research wishes to improve the reproducibility of the work that we publish. This form provides structure for consistency and transparency in reporting. For further information on Nature Research policies, see Authors & Referees and the Editorial Policy Checklist.

Statistical parameters

When statistical analyses are reported, confirm that the following items are present in the relevant location (e.g. figure legend, table legend, main text, or Methods section).

n/a	Confirmed	
☐	☒	The exact sample size (n) for each experimental group/condition, given as a discrete number and unit of measurement
☐	☒	An indication of whether measurements were taken from distinct samples or whether the same sample was measured repeatedly
☐	☒	The statistical test(s) used AND whether they are one- or two-sided<br>*Only common tests should be described solely by name; describe more complex techniques in the Methods section.*
☒	☐	A description of all covariates tested
☐	☒	A description of any assumptions or corrections, such as tests of normality and adjustment for multiple comparisons
☐	☒	A full description of the statistics including central tendency (e.g. means) or other basic estimates (e.g. regression coefficient) AND variation (e.g. standard deviation) or associated estimates of uncertainty (e.g. confidence intervals)
☐	☒	For null hypothesis testing, the test statistic (e.g. F, t, r) with confidence intervals, effect sizes, degrees of freedom and P value noted<br>*Give P values as exact values whenever suitable.*
☒	☐	For Bayesian analysis, information on the choice of priors and Markov chain Monte Carlo settings
☒	☐	For hierarchical and complex designs, identification of the appropriate level for tests and full reporting of outcomes
☒	☐	Estimates of effect sizes (e.g. Cohen's d, Pearson's r), indicating how they were calculated
☐	☒	Clearly defined error bars<br>*State explicitly what error bars represent (e.g. SD, SE, CI)*

Our web collection on statistics for biologists may be useful.

Software and code

Policy information about availability of computer code

Data collection	*Provide a description of all commercial, open source and custom code used to collect the data in this study, specifying the version used OR state that no software was used.*
Data analysis	*Provide a description of all commercial, open source and custom code used to analyse the data in this study, specifying the version used OR state that no software was used.*

For manuscripts utilizing custom algorithms or software that are central to the research but not yet described in published literature, software must be made available to editors/reviewers upon request. We strongly encourage code deposition in a community repository (e.g. GitHub). See the Nature Research guidelines for submitting code & software for further information.

Data

Policy information about availability of data

All manuscripts must include a data availability statement. This statement should provide the following information, where applicable:

- Accession codes, unique identifiers, or web links for publicly available datasets
- A list of figures that have associated raw data
- A description of any restrictions on data availability

Provide your data availability statement here.

Field-specific reporting

Please select the best fit for your research. If you are not sure, read the appropriate sections before making your selection.

☒ Life sciences ☐ Behavioural & social sciences ☐ Ecological, evolutionary & environmental sciences

For a reference copy of the document with all sections, see nature.com/authors/policies/ReportingSummary-flat.pdf

Life sciences study design

All studies must disclose on these points even when the disclosure is negative.

Sample size	To calculate sample size we performed a G-Power analysis on data gathered with CAIA and CIA to determine necessary sample size. We included parameters as means and calculate effect size with a two-tailed test. The sample size of the mice experiments was not altered during the experiments.
Data exclusions	They were followed during all the experiments. The only exclusion data to be mentioned are the following. For determining the incidence only scores of at least 0.5 (=inflamed toe) were considered. Similarly, data on inflammation associated bone erosion were conducted with mice that had a clinical score of ≥ 1 on the scanned hind paw under various conditions. We have no (not shown) outliers to be reported.
Replication	Each mouse experiment was repeated twice with similar outcomes. All data are from biological different samples and all in vitro experiments were repeated at least four times.
Randomization	In the CIA and CAIA mice experiments the equal age male mice were divided randomly on base of weight and initial cage number in the three different conditions (voluntary running, control and unloading). For the RAG2-/- experiments comparable aged male and female mice were used, and were randomly divided into the conditions, no differences are observed between the gender types.
Blinding	Allocation was done randomly. During the experiment mice were checked daily for healthy conditions and every two days they were scored for clinical symptoms by two experienced independent investigators. Clinical scoring was done respectively semi- and full blinded by two experienced investigators (minimal differences observed between both), all further sample analyses like H&E, bone erosion and ELISA was done blinded.

Reporting for specific materials, systems and methods

Materials & experimental systems

n/a	Involved in the study
☒	☐ Unique biological materials
☐	☒ Antibodies
☒	☐ Eukaryotic cell lines
☒	☐ Palaeontology
☐	☒ Animals and other organisms
☐	☒ Human research participants

Methods

n/a	Involved in the study
☐	☐ ChIP-seq
☐	☒ Flow cytometry
☐	☐ MRI-based neuroimaging

Antibodies

Antibodies used	All antibody information is shown in the Methods section.
Validation	Validation was done of all flow cytometry antibodies on both cells and on beats in several exploratory experiments.

Animals and other organisms

Policy information about studies involving animals; ARRIVE guidelines recommended for reporting animal research

Laboratory animals	All relevant information is shown in the methods section. CIA mice were C57BL/6 9-10 weeks old at the beginning of the experiment. TNFARE mice were all male and 6-9 weeks old when starting experiments. RAG2-/- mice were 9-10 weeks and CCL2-/- mice were 9 weeks old when starting the experiments. And finally comparable aged C57BL/6 mice used in CAIA were 10-12 weeks old when starting the experiments.
Wild animals	Noting to report

Field-collected samples | Noting to report

Human research participants

Policy information about studies involving human research participants

Population characteristics	X-Rays of feet were collected from a consecutive cohort of 63 RA patients with mean age 59.3 +- 11.55, including 19 males and 44 females. HR-qQCT imaging was done on 5 healthy controls and 10 age- and gender-matched sppondyloarthritis patients that fulfilled ASAS classification criteria. Human tendon samples were required by surgery for chronic Achilles tendinopathy, 7 independent samples were collected with a mean age of 52.7 +- 2.95 years. As control (unloaded) samples we gathered samples of patients with a hemiplegia who required a surgical Achilles tendon lengthening because of spasticity, mean age was 3.4 +- 20.7 years
Recruitment	Patients were recruited at the hospitals.

ChIP-seq

Data deposition

☐ Confirm that both raw and final processed data have been deposited in a public database such as GEO.

☐ Confirm that you have deposited or provided access to graph files (e.g. BED files) for the called peaks.

Data access links<br>*May remain private before publication.*	*For "Initial submission" or "Revised version" documents, provide reviewer access links. For your "Final submission" document, provide a link to the deposited data.*
Files in database submission	*Provide a list of all files available in the database submission.*
Genome browser session<br>(e.g. UCSC)	*Provide a link to an anonymized genome browser session for "Initial submission" and "Revised version" documents only, to enable peer review. Write "no longer applicable" for "Final submission" documents.*

Methodology

Replicates	*Describe the experimental replicates, specifying number, type and replicate agreement.*
Sequencing depth	*Describe the sequencing depth for each experiment, providing the total number of reads, uniquely mapped reads, length of reads and whether they were paired- or single-end.*
Antibodies	*Describe the antibodies used for the ChIP-seq experiments; as applicable, provide supplier name, catalog number, clone name, and lot number.*
Peak calling parameters	*Specify the command line program and parameters used for read mapping and peak calling, including the ChIP, control and index files used.*
Data quality	*Describe the methods used to ensure data quality in full detail, including how many peaks are at FDR 5% and above 5-fold enrichment.*
Software	*Describe the software used to collect and analyze the ChIP-seq data. For custom code that has been deposited into a community repository, provide accession details.*

Flow Cytometry

Plots

Confirm that:

☒ The axis labels state the marker and fluorochrome used (e.g. CD4-FITC).

☒ The axis scales are clearly visible. Include numbers along axes only for bottom left plot of group (a 'group' is an analysis of identical markers).

☒ All plots are contour plots with outliers or pseudocolor plots.

☒ A numerical value for number of cells or percentage (with statistics) is provided.

Methodology

Sample preparation	Samples were freshly collected from mice and processed immediatedly. Human samples were freshly collected and stored in medium for a limited time periode, whereafter they were processed. Samples were digested with collagenases to be able to get single cell solution for flow cytometry.
Instrument	BD FACS Canto II
Software	FLOWJO versie 9

Cell population abundance	For mouse data: CD11b+ populations range from 5000-110000 in tendon and 40000-500000 in synovium samples. Classical monocytes in diseased tendon samples showed levels from 100-3000, synovium samples showed levels of 500-8000 cells. For human data CD45+ cells were around 8000 in healthy and 23000 in pathogenic samples, CD11b+ were around 4000 in healthy and 19000 in pathogenic samples. CD14+ cells were around 3500 in healthy and 18000 in pathogenic samples.
Gating strategy	Boundaries between positive and negative are defined on base of gating in positive samples and 10^3 threshold. For mouse samples: after doublets were removed dead cells were excluded by use of DAPI, starting from those DAPI- singlet cells we determined NK cells by NK1.1 positive cells. In the lymphocyte gate we seperated T cells from B cells by use of respectively CD3e and CD19. Further we used all cells except CD19+, CD3e+ and NK1.1+ cells, these cells were gated by use of CD11b and CD11c markers. CD11b+ cells were divided by the F4/80 marker (macrophages) or by the Ly6G marker identifying neutrophils. Ly6G- cells were further separated by use of Ly6C to identify classical monocytes (SSC-A low and LY6C ++), patrolling monocytes (SSC-A low and Ly6C low), intermediate monotyes (SSC-A low Ly6C+) and fibroblasts. For human samples: Gating was started by removing the doublet cells. The alive cells were gated from the DAPI negative cells and used for further analysis. The hematopoietic cell population was gated in the CD45+ population, leaving out the stromal cells. Out of this the CD11B population was selected. Out of this population the CD14++and CD16+ positive cells could be demonstrated.

☒ Tick this box to confirm that a figure exemplifying the gating strategy is provided in the Supplementary Information.

Magnetic resonance imaging

Experimental design

Design type	Not applicable
Design specifications	*Specify the number of blocks, trials or experimental units per session and/or subject, and specify the length of each trial or block (if trials are blocked) and interval between trials.*
Behavioral performance measures	*State number and/or type of variables recorded (e.g. correct button press, response time) and what statistics were used to establish that the subjects were performing the task as expected (e.g. mean, range, and/or standard deviation across subjects).*

Acquisition

Imaging type(s)	*Specify: functional, structural, diffusion, perfusion.*
Field strength	*Specify in Tesla*
Sequence & imaging parameters	*Specify the pulse sequence type (gradient echo, spin echo, etc.), imaging type (EPI, spiral, etc.), field of view, matrix size, slice thickness, orientation and TE/TR/flip angle.*
Area of acquisition	*State whether a whole brain scan was used OR define the area of acquisition, describing how the region was determined.*

Diffusion MRI ☐ Used ☒ Not used

Preprocessing

Preprocessing software	*Provide detail on software version and revision number and on specific parameters (model/functions, brain extraction, segmentation, smoothing kernel size, etc.).*
Normalization	*If data were normalized/standardized, describe the approach(es): specify linear or non-linear and define image types used for transformation OR indicate that data were not normalized and explain rationale for lack of normalization.*
Normalization template	*Describe the template used for normalization/transformation, specifying subject space or group standardized space (e.g. original Talairach, MNI305, ICBM152) OR indicate that the data were not normalized.*
Noise and artifact removal	*Describe your procedure(s) for artifact and structured noise removal, specifying motion parameters, tissue signals and physiological signals (heart rate, respiration).*
Volume censoring	*Define your software and/or method and criteria for volume censoring, and state the extent of such censoring.*

Statistical modeling & inference

Model type and settings	*Specify type (mass univariate, multivariate, RSA, predictive, etc.) and describe essential details of the model at the first and second levels (e.g. fixed, random or mixed effects; drift or auto-correlation).*
Effect(s) tested	*Define precise effect in terms of the task or stimulus conditions instead of psychological concepts and indicate whether ANOVA or factorial designs were used.*

Specify type of analysis: ☐ Whole brain ☐ ROI-based ☐ Both

Statistic type for inference (See Eklund et al. 2016)	*Specify voxel-wise or cluster-wise and report all relevant parameters for cluster-wise methods.*

Correction

Describe the type of correction and how it is obtained for multiple comparisons (e.g. FWE, FDR, permutation or Monte Carlo).

Models & analysis

n/a	Involved in the study
☒	☐ Functional and/or effective connectivity
☒	☐ Graph analysis
☒	☐ Multivariate modeling or predictive analysis
